# Dynamic Behavior of Droplet Impact on Inclined Surfaces with Acoustic Waves

**DOI:** 10.1021/acs.langmuir.0c01628

**Published:** 2020-08-07

**Authors:** Mehdi H. Biroun, Mohammad Rahmati, Ran Tao, Hamdi Torun, Mehdi Jangi, Yongqing Fu

**Affiliations:** † Faculty of Engineering and Environment, Northumbria University, Newcastle upon Tyne NE1 8ST, U.K.; ‡ Shenzhen Key Laboratory of Advanced Thin Films and Applications, College of Physics and Optoelectronic Engineering, Shenzhen University, Shenzhen 518060, China; § Department of Mechanical Engineering, University of Birmingham, Birmingham B15 2TT, U.K.

## Abstract

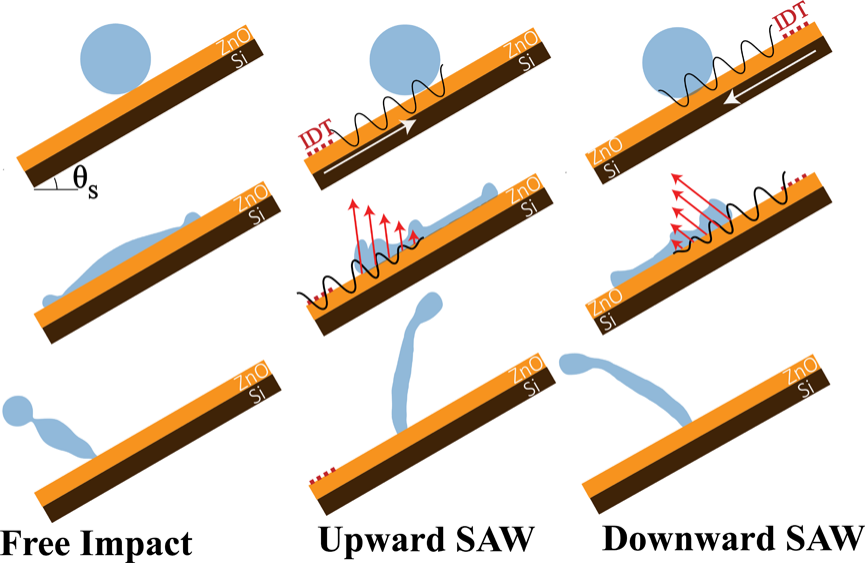

Droplet impact on arbitrary inclined surfaces is of great interest for applications such as antifreezing, self-cleaning, and anti-infection. Research has been focused on texturing the surfaces to alter the contact time and rebouncing angle upon droplet impact. In this paper, using propagating surface acoustic waves (SAWs) along the inclined surfaces, we present a novel technique to modify and control key droplet impact parameters, such as impact regime, contact time, and rebouncing direction. A high-fidelity finite volume method was developed to explore the mechanisms of droplet impact on the inclined surfaces assisted by SAWs. Numerical results revealed that applying SAWs modifies the energy budget inside the liquid medium, leading to different impact behaviors. We then systematically investigated the effects of inclination angle, droplet impact velocity, SAW propagation direction, and applied SAW power on the impact dynamics and showed that by using SAWs, droplet impact on the nontextured hydrophobic and inclined surface is effectively changed from deposition to complete rebound. Moreover, the maximum contact time reduction up to ∼50% can be achieved, along with an alteration of droplet spreading and movement along the inclined surfaces. Finally, we showed that the rebouncing angle along the inclined surface could be adjusted within a wide range.

## Introduction

Over the past decades, liquid droplet impact on solid surfaces, on either flat, inclined, or complex-shaped surfaces, has been extensively studied because of its significance in scientific understanding and industrial applications, including antifogging,^1^ antiacing,^2−4^ inkjet printing,^5−8^ agriculture,^9,10^ spray cooling,^11,12^ self-cleaning,^13−15^ anticorrosion,^16−18^ internal combustion engines,^19,20^ optical devices,^21^ anti-infection surfaces,^22^ water collection systems,^23,24^ and liquid material transportation and distribution.^25,26^

After the droplet impact on solid surfaces (either horizontal or inclined surfaces) and in the absence of splashing, the droplet spreads on the solid surface to a maximum spreading diameter, and then depending on the surface and liquid physiochemical properties and impact velocity, the droplet can retract or permanently remain spread on the surface.^27^ The droplet impact is controlled by kinematic, surface, and potential energies and viscous dissipation in the liquid medium.^28^ When the solid surface is hydrophobic, less energy is dissipated during the impact, and droplet detachment from the surface as a jet can often be observed.^29^ Experimental studies from Bayer and Megaridis have shown that the wetting properties of the surface affect the contact line velocity, capillary waves on the liquid–gas interface during the early stages of the impact, contact angle hysteresis, and the impact regime of the droplet.^30^

In the last two decades, the droplet impact dynamics on the inclined surfaces have been investigated in detail, using high-speed photography and advanced numerical methods. For instance, Šikalo et al. investigated the effects of surface roughness and liquid viscosity on the dynamics of the droplet impact on inclined surfaces. They reported the observation of asymmetry in the front and back sides of the droplet after the impact.^31^ A few studies have attempted to explain the main contributing parameters in the droplet impact regime on the inclined surfaces. For example, Bird et al. reported that the tangential velocity vector plays a major role in the droplet splash dynamics on inclined surfaces.^32^ Chiarot and Jones^33^ and Zheng et al.^34^ showed that the rebouncing regime of the high-velocity impact of continuous droplet stream on inclined superhydrophobic surfaces is functions of droplet ejection rate and impact velocity.

Moreover, different key parameters affecting the suppression of droplet splash on inclined surfaces were systematically investigated by Hao et al.^35^ Yeong et al. also investigated the correlation between the parameters of impact dynamics on inclined surfaces (such as contact time and impact regime) and the Weber number (*We* = ρ_l_*U*_0_^2^*D*_0_/γ_LV_ in which ρ_l_, *U*_0_, *D*_0_, and γ_LV_ are density, impact velocity, initial diameter, and surface tension of the droplet correspondingly).^36^ Antonini et al. observed six different rebouncing regimes, according to *We* numbers and superhydrophobic conditions.^37^ LeClear et al. observed the transition from the Cassie–Baxter impact to the Wenzel impact during the droplet impact on tilted superhydrophobic surfaces.^38^ Wang et al. showed that by increasing the inclination angle or impact velocity, there is a noticeable contact time reduction because of asymmetric spreading and retracting of the impacting droplet.^39^

Inspired by nature, a few passive techniques have been developed and applied to reduce the droplet contact time on superhydrophobic and inclined surfaces.^40−42^ For instance, Regulagadda et al. proposed texturing the substrate with a triangular ridge to realize droplet ski-jumping from the surface, thus leading to a contact time reduction of ∼65%.^25^ Zhang et al. reported a 10–30% contact time reduction by using substrates patterned with varied posts and coated with nanoparticles for oblique droplet impact.^43^ However, the efficacy and practical fabrication and applications of these proposed methods are still controversial, and there is no report of an active method that can change the droplet impact regime, contact time, and rebouncing angle on an inclined surface for any random impact scenario.

Recently, surface acoustic wave (SAW)-based microfluidics has found many applications in biochemical analysis, lab-on-a-chip,^44^ DNA sequencing,^45,46^ disease diagnosis,^47^ and drug delivery systems.^48^ SAW is generated by applying a radio frequency (RF) signal to interdigital transducers (IDTs), which are patterned on a piezoelectric substrate such as LiNbO_3_ and zinc oxide film on a solid substrate. The amplitude of the SAW and wave frequency can be altered by changing the applied RF signal power and IDT design, respectively. When a liquid phase (i.e., a droplet or a confined liquid in a microchannel) is positioned on the SAW propagating path, it attenuates and changes the mode of SAW to leaky SAW because of the discrepancy between the sound velocities in the solid and liquid media.^49^ The leakage of the acoustic energy/pressure into the liquid medium is along the Rayleigh angle, θ_R_, given by^50^
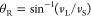
1where *v*_L_ and *v*_S_ are the wave velocities of sound in the liquid and solid, respectively. Depending on the energy transferred inside the liquid, internal streaming, transportation, jetting, and nebulization of the droplet can be generated.^51,52^ SAW-based microfluidics has the advantages of large input energy, simple device structure, fast operation, compatibility with sensing applications, and remote control, compared to other microfluidic mechanisms.

Previously, we reported that by applying traveling SAWs to a droplet during its impingement on a flat surface, the contact time could be effectively reduced.^53^ Our results showed that the transferred SAW energy into the liquid medium during the impingement can alter the internal recirculation field of the droplet, which leads to a faster detachment of droplet from the surface.

**Figure 1:**
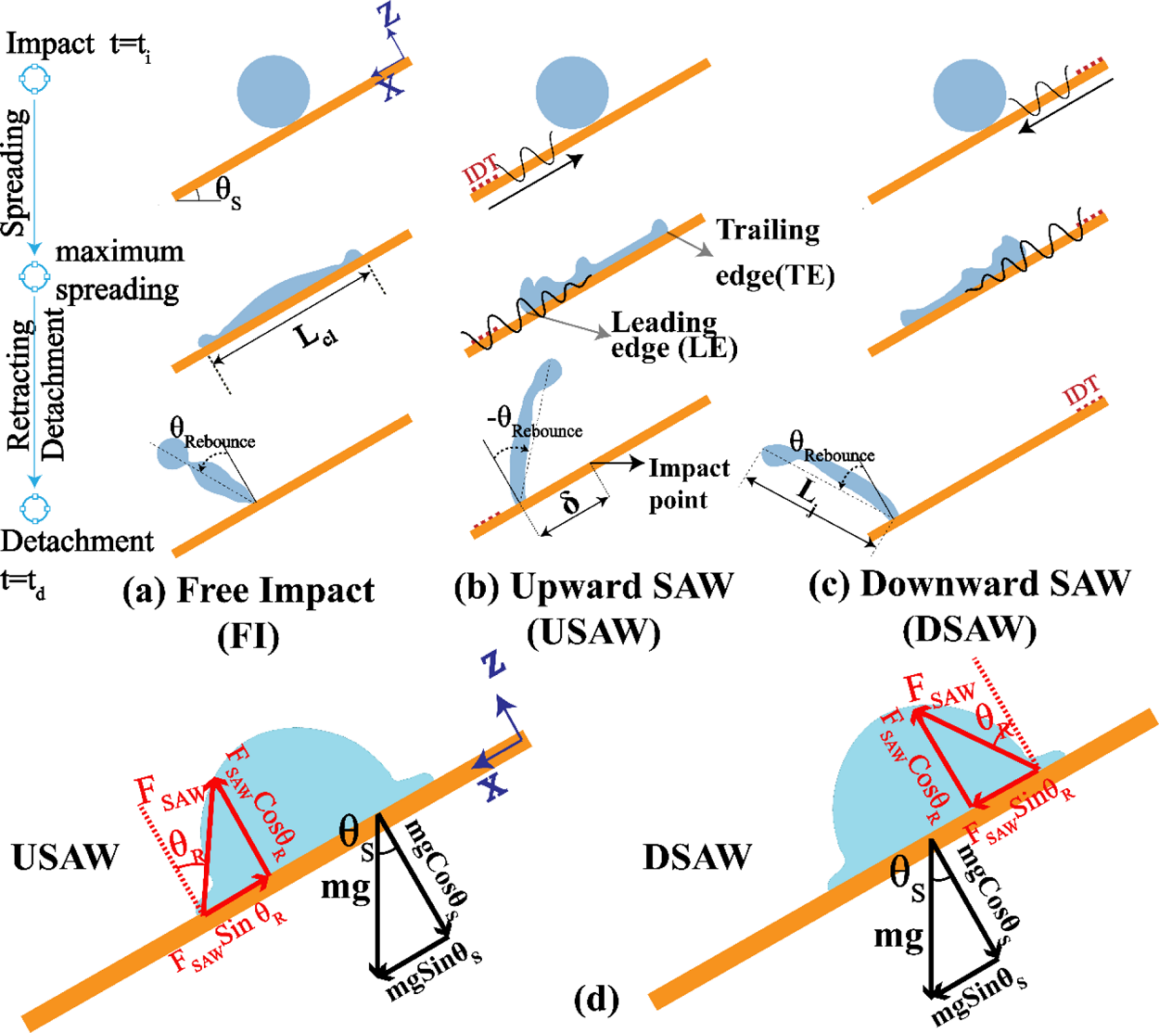
Schematic views of different scenarios of droplet impact on inclined surfaces. (a) Droplet FI, (b) droplet impact in the presence of USAW, (c) droplet impact in the presence of DSAW. The positive direction of the rebouncing angle is in the anticlockwise direction from the surface normal direction. (d) Schematic view of the SAW and gravitational force interaction.

In this work, we propose to use SAWs for the active control of droplet impact dynamics (including impact regime, contact time, and rebouncing angle) on inclined surfaces. By applying SAWs with different propagation directions and powers on inclined surfaces, the impact regime of the droplet can be effectively modified. Additionally, different impact parameters such as contact time, maximum spreading diameter, and rebouncing angle can be dramatically altered. We expect that by applying upward SAW (USAW) or downward SAW (DSAW) and as a result of changing the energy budget within the liquid medium, the motion of the droplet’s leading and tailing edges (see Figure 1 for definitions) would be altered. Consequently, the impact characteristic parameters such as contact time (which is defined as the time between impact moment, *t*_i_, and detachment moment, *t*_f_), maximum spreading (β_max_ = *L*_max_/*D*_0_, where *D*_0_ is the initial droplet diameter, and *L*_max_ is the maximum spreading width along the direction tangential to the surface), movement along the surface (δ is the distance between the impact and detachment points), and rebounce angle (θ_rebounce_, which is defined as the angle between the surface normal vector and the line connecting the separation point to the droplet tip at the separation moment in an anticlockwise direction) could be altered in a programmable and controllable way. Definitions of all these parameters are illustrated in Figure 1.

To examine the effect of SAW on the droplet impact, we propose three scenarios, for example, droplet-free impact (FI), droplet impact in the presence of USAW, and DSAW on the inclined surface, as illustrated in Figure 1a–c. To study the transferred energy of the SAW to the liquid phase, we assume that a body force, ***f***_SAW_, is generated by the SAWs and applied to the droplet along the Rayleigh angle
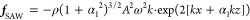
2where the symbols in bold are used to indicate the vector and tensor variables. In this equation  is the attenuation coefficient, *A* is the wave amplitude, ω is the angular frequency, and *k* is the leaky SAW wavenumber.^54^*x* and *z* are the tangential and normal positions based on the origin of the coordinate at the incidence point of the SAW and droplet on the device surface.

By changing the surface inclination angle, SAW power, and direction, the force balance between the tangential and normal components of the applied SAW force and gravitational force are changed (see Figure 1d and Supporting Information S1). For the FI scenario, after the droplet impact on solid surfaces, the droplet spreads to a maximum spreading width and then retracts toward the center. During the droplet impingement on the solid surface, two main forces along the surface resist against the liquid motion: (1) a pinning force that is generated along the three-phase contact line (TPCL) because of the contact angle hysteresis and is a function of liquid surface tension, TPCL length, and receding and advancing contact angles (e.g., , where γ_LV_ is the surface tension coefficient, *D* is the TPCL length, and θ_rec_ and θ_adv_ are the advancing and receding contact angles of the droplet on the solid surface, respectively^55,56^) and (2) the friction between the liquid and solid surfaces because of the shear stress, which is a function of the viscosity of the liquid and the relative velocity between the fluid and surface.^57^ If the initial energy of the droplet is high enough to overcome the energy dissipated by these two forces and viscous dissipation within the liquid medium, the droplet can detach from the surface at the end of the retracting phase. The interaction between the resistive forces and the gravitational force would be altered by applying the SAW force. Moreover, as SAW energy is applied to the liquid medium during the impingement, the energy budget of the droplet can be effectively modified.

To investigate our hypotheses and reveal the complex physics behind the SAW effects on droplet impact, we performed numerical simulations for the defined scenarios using a coupled level set volume of fluid (CLSVOF) finite volume method. Afterward, we experimentally examined the droplet impact dynamics in the presence of SAWs. To quantitively compare the effect of SAW on the impact dynamics, impact characteristic parameters such as contact time, maximum spreading width, droplet transition along the surface, and rebounce angle were analyzed as the functions of SAW power and direction, surface inclination angle, and impact velocity.

Our results show that at a constant *We* number, by increasing the applied SAW power, regardless of the SAW direction and surface inclination angle, the contact time of the impacting droplet can be reduced. Additionally, the impact regime can be changed from deposition (in the FI scenarios) to a complete rebound by applying SAW agitation. More interestingly, if the surface inclination angle is kept constant and the impact velocity (i.e., the *We* number) is altered, the impact regime at the lower *We* numbers can be changed from deposition on the surface to complete rebound from the surface.

## Experimental Methods and Sample Preparation

### SAW Device Preparation

Using a direct current (DC) magnetron sputter system (Nordiko Ltd.), a layer of ZnO piezoelectric film with a thickness of ∼5.5 μm was deposited on Si substrates using a pure zinc target (99.99%). The deposition parameters are as follows: a DC power of 400 W, an Ar/O_2_ mass flow ratio of 10/15 sccm, and a chamber pressure of ∼3.2 mTorr without any external substrate heating. SAW devices were fabricated on a ZnO film-coated silicon wafer [see Supporting Information Figure S3 for a scanning electron microscopy (SEM) image of the film/substrate], on which two pairs of IDTs were photolithographically patterned. The Cr/Au IDTs had thicknesses of 20/100 nm and consisted of 30 pairs of fingers, with an aperture of 5 mm and different wavelengths of 64–200 μm. The resonant frequency of each SAW device was measured using an RF network analyzer (HP 8752A RF network analyzer). The SAW device surface was coated by a layer of CYTOP (Asahi Glass Co.) with a thickness of ∼200 nm. The droplet contact angle was measured to be 122° ± 2°, with a contact angle hysteresis of 28° ± 6°. The RF signal was generated using a signal generator (Macroni2024) and amplified with an RF amplifier (Amplifier Research, 75A250) before being applied to the IDTs of the SAW device. The power applied to the SAW IDTs was measured before each experiment using an RF power meter (RACAL Equipment, 9104).

### Droplet Impact

Droplets of deionized water with an initial diameter of *D*_0_ = 1.9 × 10^–3^ m were generated from hypodermic needles (BD Microlance, inner diameter *D*_n_ = 1.5 × 10^–3^ m) mounted on a 2D positioner using a syringe pump (Cellix, World Precision Instruments, UK). The calculation of the droplet volume was based on the numerical model proposed by Aminzadeh et al.^58^ The droplets were released from differently selected heights, *H*, with an initial velocity of zero to reach the desired velocities before their impacts on the inclined solid surface. The inclination angle of the device surface was set to be 0, 15, 30, 45, and 60°. The impact and rebouncing sequences were captured from a side view using a high-speed camera (HotShot 1280CC) with a macro lens (120 mm BRAND) at 5000 frames per second and a resolution of 432 × 244 pixels. MATLAB image processing tool was used to calculate the impact velocity of the droplet from two consecutive images just before its impact onto the device surface. To fully understand how the SAW can modify the droplet impact on inclined surfaces, a set of systematic experiments was performed to investigate the effects of inclination angle, impact velocity, and SAW direction and power, at a lab temperature of 21 ± 0.5 °C and 50 ± 5% relative humidity. Under this temperature, the density and surface tension of the deionized (DI) water are 995 kg·m^–3^ and 0.072 N·m^–1^, respectively. To confirm the repeatability of the experiments, each test was repeated four times.

### Uncertainty Analysis

The diameter of the dispensing needle (*D*_n_ = 3 × 10^–4^ m) was captured and measured, and the data were used to calibrate the images. A conversion factor of 40 μm/pixel was obtained. The resolution of the optical imaging system for observing the droplets in our system was determined to be 120 μm based on edge detection methods corresponding to three pixels. On the other hand, the repeatability of the droplet diameter and impact velocity should be examined. Figure S3a in the Supporting Information shows that the uncertainty of the droplet diameter was ±3.8%. In principle, the impact velocity can be calculated by the equation, . The results of Figure S3b in the Supporting Information shows that the uncertainty of the impact velocity was ±4.5%. The value of the relative error of *We* number was calculated by the equation, Δ*We*/*We* = Δ*D*_0_/*D*_0_ + 2Δ*U*_0_/*U*_0_ to be 12.8%.^59^ The angle deviation of the SAW device holder was ±0.3°.

## Results and Discussion

### Impact Mechanism Based on Numerical Simulations

**Figure 2:**
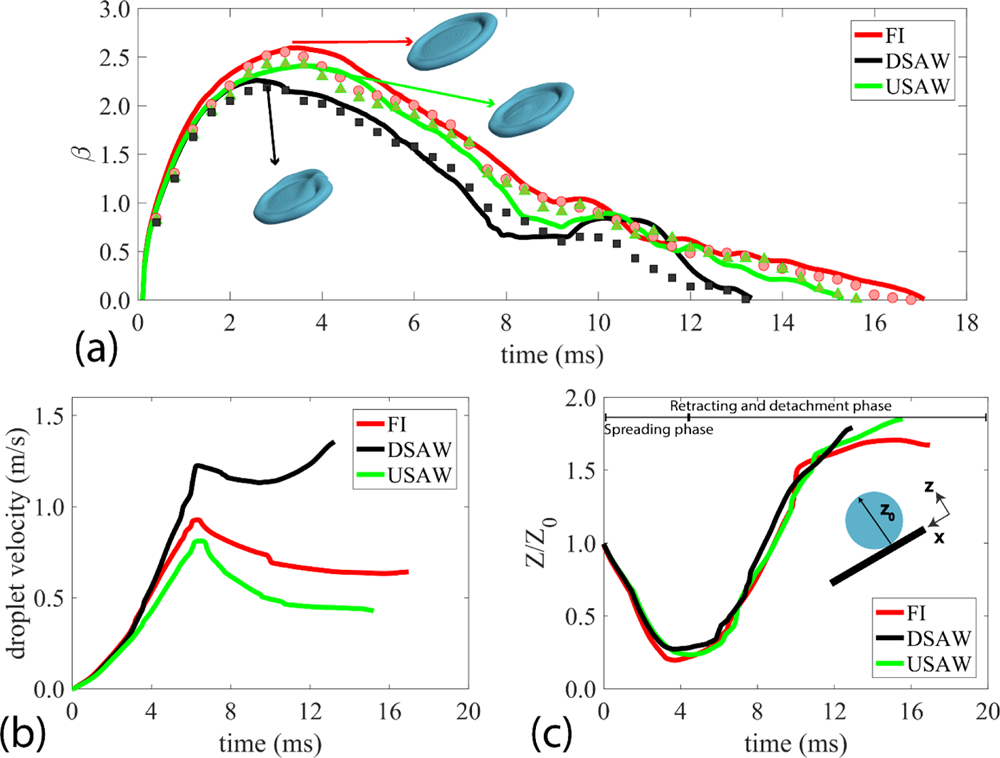
(a) Quantitative comparison between the simulation and experimental results for the droplet contact width evolution. (b) Temporal droplet velocity (i.e., an average of leading- and tailing-edge velocities) in the *X*-direction. (c) Temporal evolution of the normalized droplet tip height in *Z*-direction.

First of all, we simulate the impact and bouncing dynamics of a spherical droplet on a solid surface with an inclination angle of 30° in three cases for FI, USAW, and DSAW scenarios. For all the simulation cases, the droplet volume and impact velocity are kept constant at 3.5 μL and 1.4 m/s, respectively. The details of the mathematical model (developed in OpenFOAM 4.x CFD toolbox), contact angle modeling, and numerical setup are presented in the Supporting Information S4–S6. To validate the numerical results, a set of experiments with the same parameters was performed (the selected examples of the results for the three cases are presented in Supporting Information Videos S1–S3). A quantitative comparison between the experimental and simulation results for the droplet contact width during the impact is shown in Figure 2a. A good agreement between the experimental and numerical results can be found, proving that simulation results can be precisely used to analyze the effect of the SAW on droplet impact. Moreover, to qualitatively validate the numerical findings, comparisons between the droplet interfaces from both the numerical and experimental results are presented in Supporting Information Figure S4. Clearly, both the quantitative and qualitative comparisons show that the developed numerical method is capable of capturing the interaction between the acoustic waves and liquid medium and also the TPCL movements.

As shown in Figure 2a, the droplet continuously spreads to its maximum diameter on the inclined surface for all the three scenarios, and then the thickened rim starts to retract toward the center of the liquid. For the DSAW (USAW) scenario, the applied SAW energy restricts the tailing edge (leading edge) from spreading. For the FI scenario, the contact width reduces until the droplet is separated from the surface after 16.8 ms. By applying DSAW, the maximum spreading width and the time to reach this width are reduced (i.e., 2.4 ms compared to 3.6 ms for the USAW and FI cases). After reaching the maximum spreading width, the contact width is gradually reduced until 8 ms after the onset of the impact. Then, it stays nearly constant for ∼2.6 ms as the droplet moves on the inclined surface. Afterward, the contact width is reduced sharply until the droplet is separated from the surface after 13.6 ms. For the USAW case, during the whole retracting phase, the contact width is lower (higher) than that in the FI (DSAW) case. Moreover, the sharp reduction in spreading width is not observed at the end of the retracting phase for the USAW scenario.

Figure 2b shows the average droplet velocity along the *X*-direction for the three cases. During the spreading phase, the velocities do not show considerable differences. After ∼2.8 ms from the onset of impact, the droplet in the DSAW scenario starts to accelerate much faster than the other two scenarios. In general, as the applied DSAW (USAW) energy promotes (restricts) the droplet motion in the *X*-direction, the droplet has a higher (lower) average velocity compared to that in the FI scenario. The ratio of the droplet tip height (highest point in *Z*-direction in liquid medium), *Z*, to its initial value, *Z*_0_ (see Figure 2c), shows that the droplet tip heights have a rather similar behavior during the impact. However, as the maximum spreading diameter of the droplet is larger for the FI scenario compared to those of USAW and DSAW scenarios, the tip position of the liquid is lower for this case at its maximum spreading.

**Figure 3:**
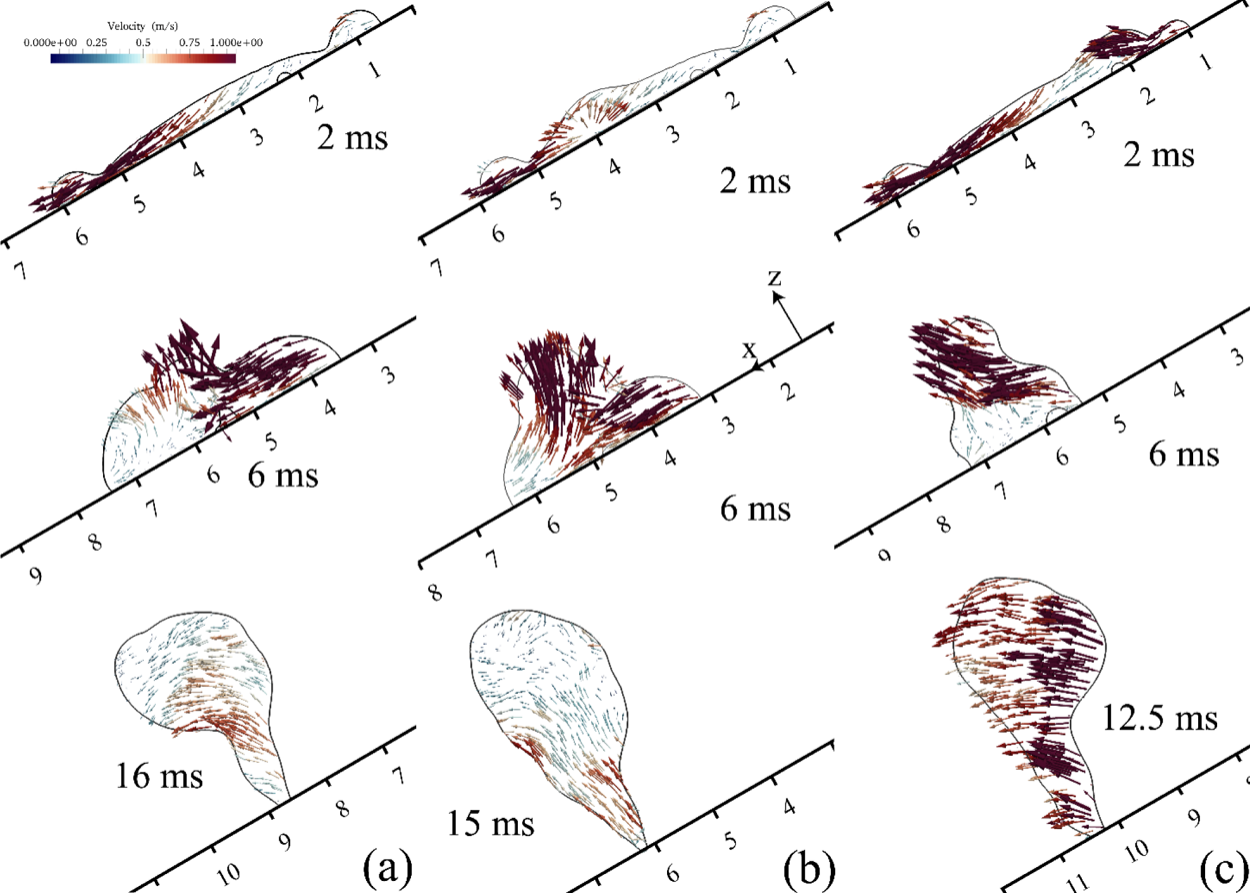
CFD snapshots of droplet interface overlaid by velocity vectors at the spreading, retracting, and detachment stages for (a) FI scenario, (b) USAW scenario, and (c) DSAW scenario. For all the cases, a droplet with a volume of 3.5 μL and *We* number of 50 has an impact on a surface with the inclination angle of 30°.

We then focus on the internal streaming patterns inside the liquid medium during the impingement for the three designed scenarios. Snapshots of internal streaming patterns in the middle plane of the droplet are illustrated in Figure 3. For the FI case, 2 ms after the onset of the impact, there is a strong velocity field in the region close to the leading edge. However, because of the viscous dissipation, this velocity field is not apparent in the tailing edge, as shown in Figure 3a. After 6 ms, the leading edge has moved ∼1.4 mm on the inclined surface, whereas the tailing edge has moved as large as 3 mm, thus resulting in a significant internal flow generation in the tailing-edge area (see Figure 3a). After 16 ms, near the last moment of the impingement, the droplet contact width is minimized, and the internal streaming pattern is faded compared to previous snapshots.

By applying USAW, after 2 ms from the onset of the impact, a hunch is noticeable in the center of the spreading droplet on the inclined surface. As the USAW restricts the droplet to spread downward, the velocity field in the leading-edge area is much weaker when compared to that of the FI case. After 6 ms, the tailing edge has moved ∼2.6 mm. In the liquid medium close to the tailing edge, a velocity field along the *X*-direction is generated, whereas near the center of the droplet, as a result of the applied SAW energy, a strong streaming pattern along the *Z*-direction is observed, which can push the droplet upward. Finally, after 15 ms, the droplet is separated from the surface with a faded internal streaming pattern in the area close to the droplet tip and a rather weak internal streaming field in the droplet root, mostly along the *Z*-direction.

For the DSAW scenario (see Figure 3c), during the spreading phase, the SAW energy causes the restriction of spreading from the tailing edge, and a strong streaming pattern is created in the area close to the tailing edge. After 6 ms, the droplet tip height is 21% larger than that of the FI case (see Figure 3c), and the internal streaming pattern in the droplet root almost disappeared. However, a strong velocity field is created in the droplet tip area. After 12.5 ms, the droplet is at its final moments of impingement, and the liquid medium has a relatively strong velocity field inside.

The simulation results clearly show that the energy delivered by SAW has changed the internal recirculation patterns upon the droplet impinging onto the inclined surfaces. By applying the USAW, in all the stages of the impact, the velocity field (especially in the leading-edge area) is slightly rotated toward the *Z*-direction. However, the intensity of the internal streaming patterns looks similar to that of the FI scenario. On the other hand, in the DSAW scenario, it is apparent that the liquid medium has a much stronger internal recirculation pattern during the impact.

To quantitively analyze the effect of the applied SAW energy, we further investigate the energy budget during the impact of the designed scenarios. During the droplet impact onto the inclined surfaces, gravitational, surface, and kinetic energies within the droplet are continuously converted among each other. Moreover, these energies are dissipated by liquid viscosity, wave generation at the gas–liquid interface, the interaction between solid and liquid phases, and subunit droplet separation.^28,60^ To reveal the physical differences among the above three scenarios, we analyze the kinetic energy, *K*, surface energy, *S*, gravitational energy, *P*, applied SAW energy, *E*_SAW_, and energy dissipation by viscosity, *E*_dis_, obtained from the numerical simulations. The kinetic energy of the droplet can be defined as the volume integral of the kinetic energy of the infinitesimal volume element, *V*, within the liquid medium
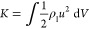
3where *u* is the magnitude of the liquid velocity. The surface energy *S* is given by
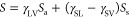
4where *S*_a_ and *S*_s_ are the areas of the droplet in contact with the gas and solid media, respectively. γ_SV_ and γ_SL_ are the surface tensions of the solid surface and solid–liquid interface. Gravitational energy, *P*, is defined based on the distance of each element in the *Z*-direction from the solid surface, *z*, and is calculated from
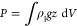
5

**Figure 4:**
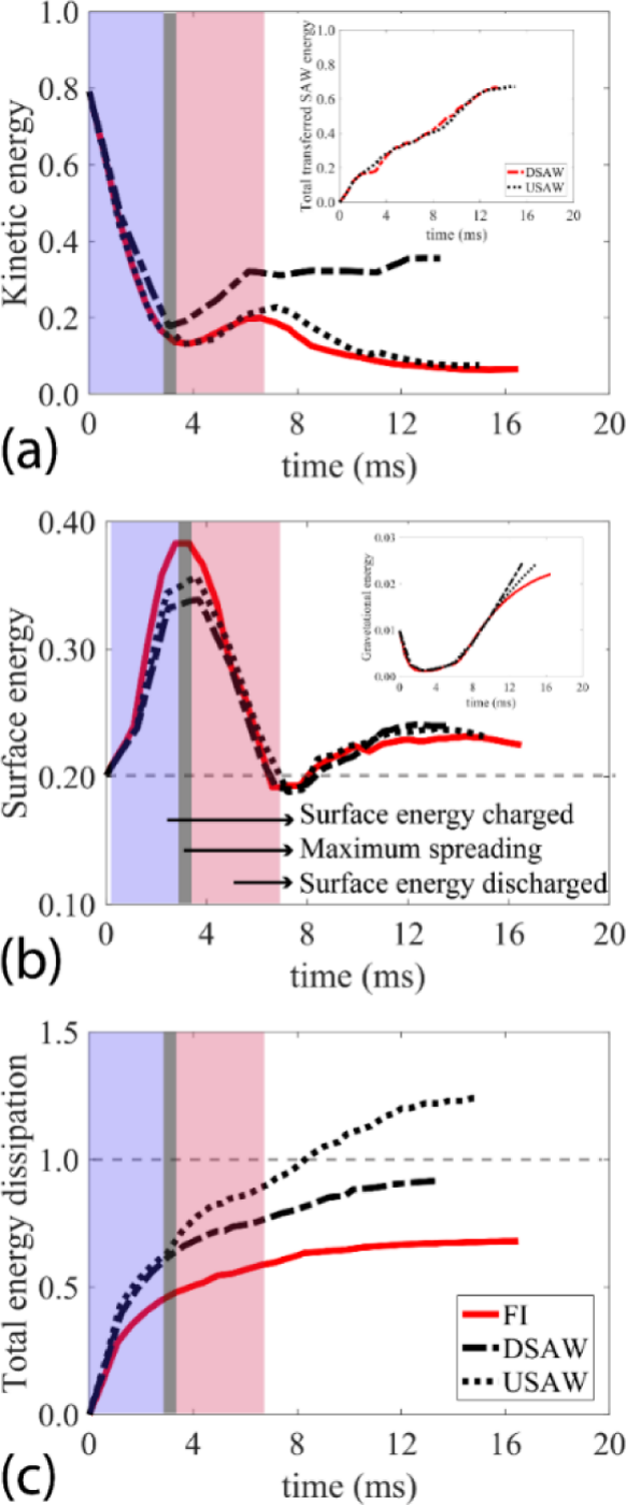
Simulation results of the effect of SAW on the temporal evolution of energy. (a) Normalized kinetic energy of the liquid medium. The embedded graph represents the total energy of the droplet. (b) Normalized surface energy of the liquid. Normalized gravitational energy is presented in the embedded graph. (c) Energy dissipation during the impingement. Blue and red areas represent the droplet spreading and retracting, respectively. All the energies are normalized with the total droplet energy at the onset of the impact.

The total energy dissipation by liquid viscosity and the applied SAW energy to the liquid medium can be defined as
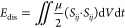
6
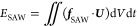
7where,  is the strain tensor.^61^ The results of the evolution of energies for the three simulated scenarios are presented in Figure 4. All the energies in Figure 4 are normalized by the initial energy of the droplet at the onset of the impact (e.g., *E*_0_ = 1/2ρ_l_*V*_0_*v*_impact_^2^ + γ_LV_*A*_0_, where *V*_0_, *v*_impact_, and *A*_0_ are the volume, velocity, and surface of the droplet at the impact moment). The results in Figure 4b show that the gravitational energy occupies less than ∼2% of the total energy during the impingement for all the cases; therefore, it is not considered in the following analysis. Figure 4c illustrates the total energy dissipation from the impact moment.

For the FI scenario at the onset of the droplet impact, 79% of its total energy is in the form of kinetic energy, and during the spreading phase (e.g., the blue area in Figure 4a–c), the kinetic energy is converted to the surface energy or dissipated by vicious and capillary dissipation. During the droplet spreading, the surface energy is increased by 18, and ∼44% of the energy of the system is dissipated. At the end of the spreading phase, there is a transient time when the surface energy stays almost constant. During the retraction phase, the surface energy is converted back to kinetic energy (e.g., the red area in Figure 4a–c). At the end of the retraction phase, the surface energy is decreased by 23.3% from its maximum, and the kinetic energy is increased by 7%. After ∼7 ms, the kinetic energy of the droplet starts to decrease because of the energy dissipation, and the droplet is separated from the surface after ∼16.8 ms.

By applying the DSAW, the *x*-component of the SAW force along the inclined surface prevents the tailing edge from spreading, and thus the maximum surface energy is ∼4.2% lower than that in the FI case. On the other hand, during the impingement, the total SAW energy, which is transferred to the liquid medium (as shown in the small graph in Figure 4a), is as much as 66% of the initial energy of the droplet. The kinetic energy of the droplet at the end of the spreading phase is decreased to ∼20%, and the energy dissipation is ∼60%. However, as a result of the applied SAW energy and conversion of the surface energy, the kinetic energy of the droplet starts to increase sharply. Once all the energy stored as surface energy is converted back to kinetic energy (i.e., after ∼8 ms), the kinetic energy stays almost constant, meaning that the applied SAW energy is dissipated during this period. Because of the relatively higher kinetic energy after the spreading stage, the droplet detaches from the surface after ∼13.6 ms, which is 20% shorter in contact time than that in the FI case.

For the USAW case, during the 6 ms after the onset of the impact, the kinetic energy has a rather trend similar to that of the FI scenario. Nevertheless, between 6 and 10 ms after the impact, the kinetic energy of the droplet is ∼5% higher on average than that of the FI case. The results indicate that despite the applied SAW energy to the droplet for both USAW and DSAW scenarios being equal (see the embedded graph in Figure 4a), the droplet gains less kinetic energy in the USAW case because of significant energy dissipation. This can be explained by the fact that the *x*-component of the SAW force is in the reverse direction of the component of gravitational force, and thus (as a result of interaction between these forces in a 3D pattern) a rather strong internal recirculation field with vortices is generated within the liquid medium, thus dissipating more energy. Interestingly, for the USAW case, the amount of dissipated energy by viscosity is higher than the initial droplet energy. The ratio of the total dissipated energy for the USAW and DSAW cases, *E*_disUSAW_/*E*_disDSAW_, is ∼1.35. This result is significant as it shows that by changing the direction of the SAW propagation, energy dissipation within the liquid and the kinetic energy of the droplet can be modified.

By comparing the simulation results from the above three scenarios on the inclined angled plate, we can conclude that by changing the direction of the applied SAW, the amount of kinetic energy and energy dissipation during the impingement can be altered to control impact parameters such as contact time and droplet movement on the surface during the impact. After understanding the physics behind the effect of SAW on the droplet impact on the inclined surface, we then experimentally investigated the effects of SAWs on the impact dynamics.

### Droplet Impact Phenomena from Experimental Results

**Figure 5:**
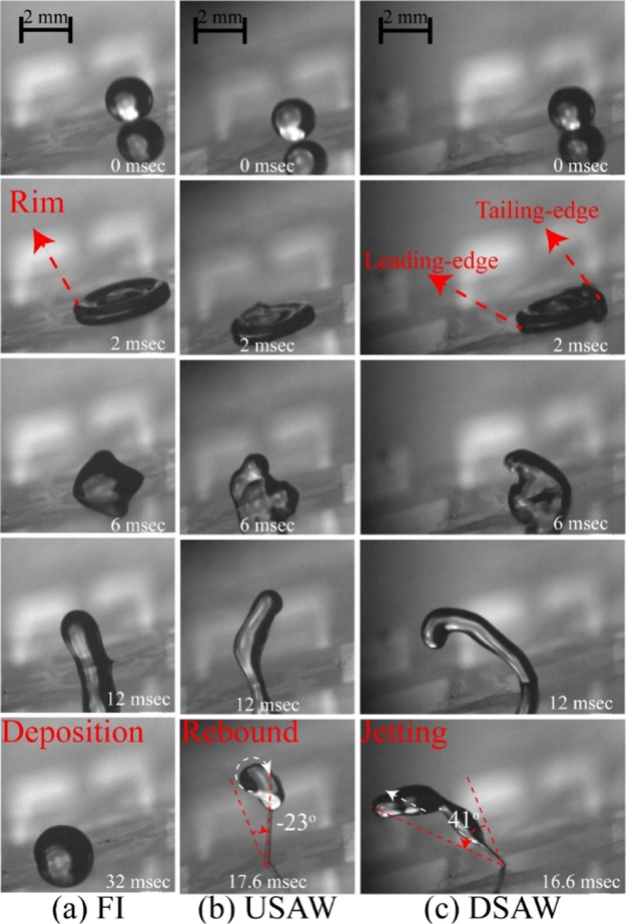
Sequential snapshots of a water droplet impacting on the solid surface with an inclination angle of 15° and a Weber number of 30.3 for (a) FI scenario, (b) USAW scenario with the power of 15 W applied to the IDTs, (c) DSAW with the power of 15 W applied to the IDTs. In all the scenarios, DI water droplet with a volume of 3.5 μL has an impact on the hydrophobic surface of the SAW device. See the Supporting Information Videos V4–V6 for the experimental movies.

Figure 5 shows snapshot examples of the impact of a droplet with a volume of 3.5 μL and a Weber number of ∼30.3 on a surface with an inclination angle of 15°, for the three designed scenarios. For the FI case (see Figure 5a), the droplet first spreads to its maximum diameter, forming a crater shape, and then the rim starts to retract toward the center. In this case, the kinetic energy of the droplet at the end of the retraction phase is not large enough to detach the whole droplet from the surface, thus leading to the deposition of the droplet after a series of vibrations on the surface.

On the other hand, from our numerical results, we know that by applying the USAWs or DSAW onto the inclined devices, the energy budget of the droplet is changed (depending on the SAW power), and correspondingly, the droplet dynamics and impact regime are changed. As shown in Figure 5b, the applied USAW deforms the leading edge of the droplet during the spreading phase, and after ∼6 ms, a liquid beam starts to form at the end of the retracting phase. As discussed in the numerical results, the USAW can slightly increase the kinetic energy of the droplet during the impingement process. As a result, the droplet is detached from the surface after ∼18 ms in a rotating sphere shape (see Figure 5b). More interestingly, by applying DSAW, the tailing edge is deformed during the spreading phase, and the kinetic energy of the liquid is intensively increased, leading to a liquid beam formation after ∼10 ms. The enhanced jet is separated from the surface along a rebouncing angle of 41° after ∼17 ms. The time evolution plot of the droplet contact line width is illustrated in Supporting Information Figure S6a. The comparisons between these three cases show that by applying the SAWs, the critical parameters of droplet impact such as contact time, impact regime, and rebouncing angle can practically be modified.

### Effects of Inclination Angle on Impact Dynamics

**Figure 6:**
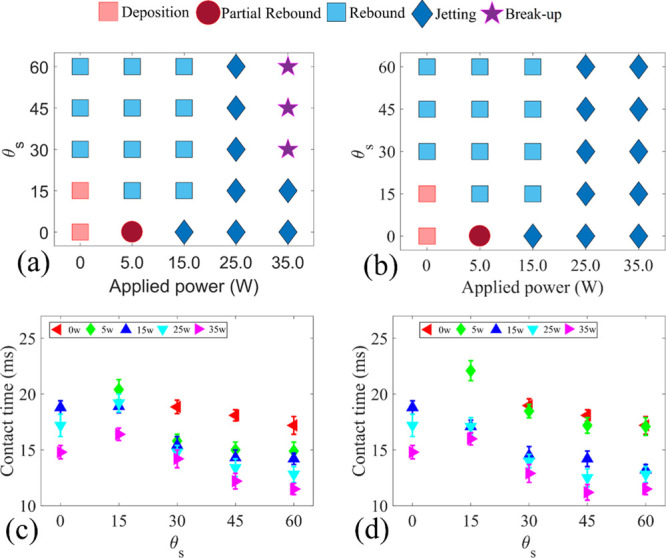
(a) Droplet impact regime map as a factor of applied SAW power and surface inclination angle for (a) USAW and (b) DSAW scenarios. Contact time versus inclination angle for different applied SAW powers for (c) USAW and (d) DSAW scenarios. Note that the contact time is not shown for the deposition and partial rebound cases. In all the cases, the droplet with a volume of 3.5 μL and the Weber number of 50 impacts the ZnO/Si SAW device.

To understand the effects of inclination angle on the impact dynamics in the presence of SAW, a set of experiments was conducted using the DI water droplets with a volume of 3.5 μL and an impact velocity of 1.4 m/s. The obtained distribution maps of the droplet impact regimes for the cases of USAW and DSAW are shown in Figure 6a,b. The impact regimes are categorized into droplet deposition, partial and complete rebound, jetting rebound, and breakup (see Supporting Information Figure S7).

In the absence of SAW and at a low inclination angle (e.g., 15° and less in this study), the droplet cannot be detached from the surface after the impact. However, by increasing the inclination angle above 15°, the droplet can be fully detached from the surface in the FI scenario. As the inclination angle increases, the tangential component of the gravitational force is increased (as illustrated in Figure 1d). Accordingly, the droplet has more kinetic energy during the retracting phase, which results in the full detachment of the droplet from the surface.

By applying SAWs during the droplet impact on inclined surfaces, the impact regime can be changed among rebound, jetting, or droplet breakup, with the gradual increase of applied powers. For the DSAW cases, by applying SAWs with high powers (i.e., with powers higher than 25 W applied to the IDTs), the liquid droplet is bounced off the solid surface as a thin beam. However, droplet breakup into several subunits is sometimes observed in the USAW cases at very high applied powers. In these cases, the droplet starts to break up after reaching the maximum diameter as the surface tension force cannot overcome the applied SAW momentum, which has been transferred into the liquid medium.

The corresponding contact times for the designed experiments are presented in Figure 6c,d. As discussed, for the inclination angles of 0 and 15°, the droplet stays stationary on the inclined surface at the end of the retracting phase, and thus the contact time is defined as indefinite for these cases. Nevertheless, SAWs can change the droplet impact regime from deposition to complete rebound or jetting from the surface. Moreover, by increasing the applied SAW power at each fixed inclination angle, the droplet contact time is reduced. The detailed analysis shows that the contact time can be effectively reduced by applying SAW. For instance, as shown in Figure 6d, for the surface inclination angle of 45°, by applying DSAW with the power of 35 W, the contact time can be reduced as much as 30% compared to the FI scenario. From the simulation results, we know that by applying SAWs (both the USAW and DSAW), the energy budget of the droplet is changed, and the droplet gains more kinetic energy during the retracting phase to bounce off the inclined surface.

**Figure 7:**
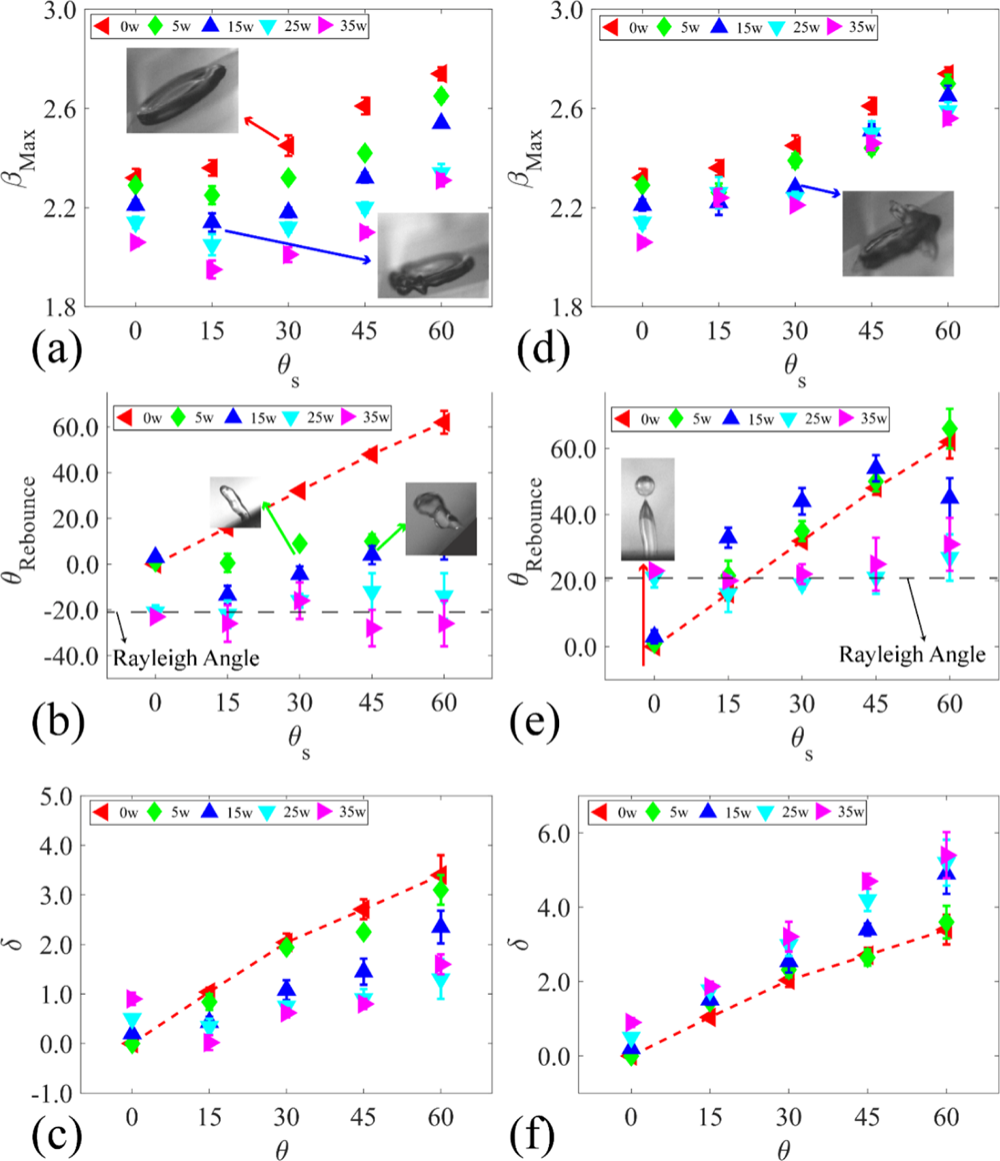
Effect of surface inclination angle on (a) maximum spreading, (b) rebounding angle, and (c) droplet movement along the *X*-direction for the USAW scenario. Effect of surface inclination angle on (d) maximum spreading, (e) rebounding angle, and (f) droplet movement along the *X-*direction for the DSAW scenario. In all the cases, the droplet with a volume of 3.5 μL and a Weber number of 50 is impacted on a ZnO/Si SAW device.

Figure 7 presents the effect of inclination angle on the maximum spreading diameter β_max_, rebounding angle, and movement along the surface. As shown in Figure 7a,d, by increasing the inclination angle for the FI scenarios, the value of β_max_ increases. For all the scenarios, by increasing the inclination angle, the tangential component of the gravitational force, *mg* sin θ_s_, enhances the spreading of the droplet front but suppresses the spreading of the back of the droplet. However, when the SAWs are applied, the tangential component of the SAW force, *f*_SAW_ sin θ_R_, limits the spreading of the droplet (e.g., the leading edge for USAW and the tailing edge for DSAW). At a lower inclination angle, the SAW force is dominated in limiting the spreading of the droplet. However, by increasing the inclination angle, the gravitational force becomes dominant, which leads to larger values of the maximum spreading diameters (see Figure S1 in the Supporting Information). The maximum spreading is reduced more by applying USAW compared to DSAW for all the angles.

The results of the droplet rebounding angles (see Figure 1 for definition) are illustrated in Figure 7b,e. In general, the interaction between the applied SAW force and the gravitational force determines the rebouncing angle of the droplet. For the FI scenarios, as the only force redirecting the droplet during rebouncing is gravity, the rebouncing angle of the droplet has a nearly linear trend as a function of inclined angle (see the dashed red line in Figure 7b,e). However, by applying lower SAW powers to the IDTs (i.e., 5–15 W), the interactions among these forces and the corresponding rebouncing angles are modified compared those of the FI cases. At higher powers, the droplet is fully detached from the surface along the Rayleigh angle. The results show that at higher powers, the SAW force is large enough compared to the gravitational force and will drive the droplet as a jet along the Rayleigh angle of the SAW device along the inclined solid surface, regardless of the inclination angle. These results show that by changing the SAW power and direction on any inclination angle, the droplet rebouncing angle can be changed. To examine the repeatability of the jet redirecting by SAW, the droplet impact with the *We* number of 50 on a surface with an inclination angle of 15° was repeated 16 times, whereas a power of 25 W was applied to the IDTs. The histogram results of the tests are presented in Figure S8 of the Supporting Information, which showed good repeatability.

Figure 7c,f shows the results of distances for droplet movements along the *X*-direction, δ, between the impact and detachment. As explained by the simulation results, for the DSAW cases, the tangential components of SAW and gravitational force tend to move the droplet forward in the *X*-direction; therefore, by increasing the SAW power or inclination angle, the value of δ increases. However, in the USAW cases, the tangential components of the gravitational and SAW forces work against each other, and the value of δ is decreased by increasing the SAW power.

### Effect of Droplet Impact Velocity

**Figure 8:**
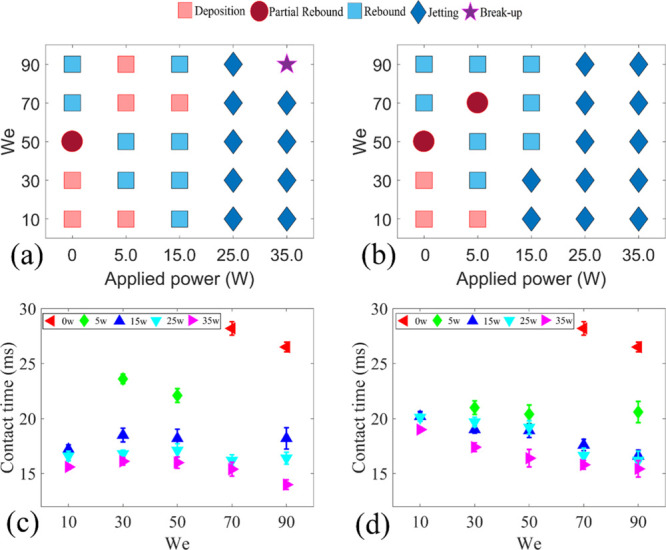
Droplet impact regime maps as functions of applied SAW power and *We* number for (a) USAW and (b) DSAW cases. Contact time vs *We* number for different applied SAW powers for (c) USAW and (d) DSAW scenarios. In all the experiments, a droplet with a volume of 3.5 μL has an impact on a ZnO/Si SAW device with an inclination angle of 15°.

Figure 8a,b presents the results of the impact regime map as a function of *We* number and SAW applied power, with the droplet volume and inclination angle of the surface fixed at 3.5 μL and 15°. For the DI cases and at lower *We* numbers (i.e., 10 and 30), the droplet stays stationary on the surface after the impact, and the contact time is indefinite. By increasing the *We* number to 50, as the initial kinetic energy of the droplet is increased, part of the droplet gains enough energy at the end of the retracting phase to be detached from the surface. However, the droplet root still stays in contact with the surface. At higher *We* numbers, the liquid kinetic energy at the end of the retracting phase is high enough to detach the whole droplet from the solid surface, so a complete rebound is observed.

For both the USAW and DSAW scenarios, jetting rebounce is observed when the applied SAW power is larger than 20 W. At these larger SAW powers, the kinetic energy induced by the SAWs is much higher than the dissipation energy because of the liquid viscosity. Correspondingly, the droplet has enough energy to be separated from the solid surface at the end of the retracting phase. Conversely, for the USAW scenario and the applied SAW power of 15 W, a complete rebound of the droplet from the surface is observed for all the *We* numbers except the *We* number of 70. This inconsistency is due to the relatively more viscous dissipation in this case, where the opposite directions of the momentums generated by gravitational and SAW forces during the spreading phase cause significant vortices within the droplet. These vortices, in turn, dissipated the kinetic energy of the droplet. Therefore, the kinetic energy of the droplet might not be high enough at the retracting phase to detach the liquid phase from the solid surface.

In order to investigate the effect of SAWs on droplet dynamics, the contact times of the droplet as a function of *We* numbers were obtained, and the results are presented in Figure 8c,d. Comparisons of these two graphs with Figure 8a,b reveal that for the impacts with *We* number lower than 70 and FI scenarios, the droplet is deposited on the surface. However, by applying SAWs with powers higher than 25 W, regardless of the SAW direction, a complete detachment of the droplet from the surface is observed. Interestingly, the results show that the contact time of the droplet can be reduced by the factor of a maximum of 48.5% by increasing the SAW power to 35 W.

**Figure 9:**
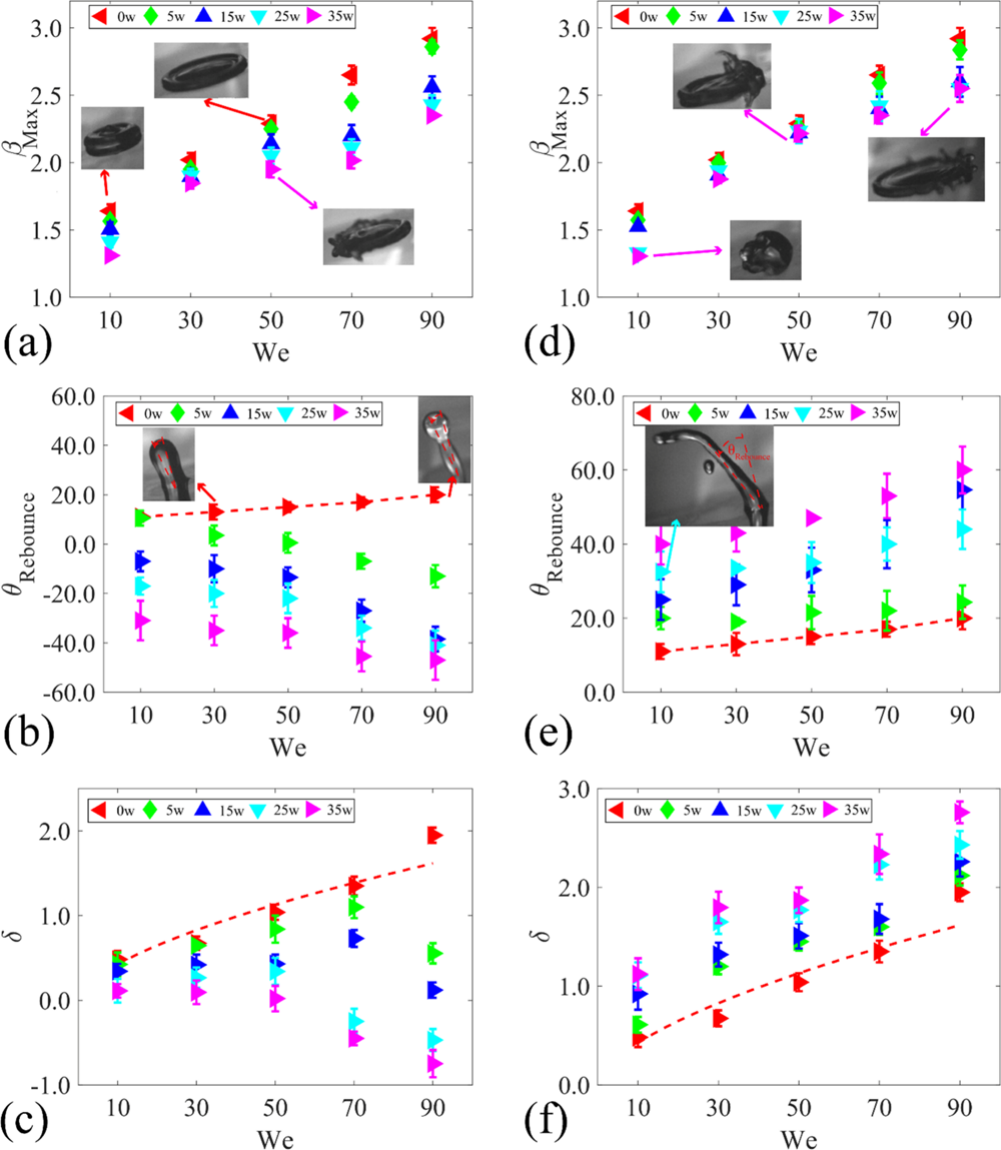
Effect of impact velocity on (a) maximum spreading, (b) rebounding angle, and (c) droplet movement along the *X-*direction for USAW scenario. Effect of impact velocity on (d) maximum spreading, (e) rebounding angle, and (f) droplet movement along the *X-*direction for DSAW scenario. In all the cases, the droplet with a volume of 3.5 μL impacts a ZnO/Si SAW device with a surface inclination angle of 15°.

Figure 9a,d shows the effects of *We* number on the maximum spreading widths of the droplet during the impact for the scenarios. Because of the increased initial kinetic energy, the value of β_max_ for FI scenarios is increased as the *We* number is increased. However, by applying the SAWs, the droplet spreading width is limited, and thus the value of β_max_ is decreased. For both USAW and DSAW scenarios, regardless of the *We* number, the maximum spreading distance is decreased by increasing the applied SAW power. This is due to the restriction of the contact line motion during the spreading phase in the area because of the applied SAWs.

The effect of *We* number on rebounding angle is illustrated in Figure 9b,e. It is apparent from these figures that by changing the SAW direction, the direction of the detached droplet is changed. For the DSAW case, as expected, by increasing the applied SAW power, the rebounding angle is increased. On the contrary, for the USAW cases, the rebounding angle is decreased significantly by changing the applied power. It is interesting to observe that a wide range of the rebouncing angle up to 110° (e.g., from −60 to 50°) can be achieved by changing the applied SAW power and direction.

Figure 9c,f shows the effect of *We* number on the values of displacement δ. For the FI scenario, with the successive increases in the *We* number, as a result of the increase in the tangential component of the gravitational force, the value of δ increases linearly. After applying the DSAW, the tangential component of the SAW force enhances the movement of the droplet in the *X*-direction, and the value of distance δ increases significantly. For the USAW scenarios, the tangential components of the gravitational and SAW forces are in opposite directions, and the interaction between these forces determines the displacement of δ values. Here, using a standard equation of δ = *AWe*^*B*^, we obtained the regression fitting for the movement of the droplet on the surface in FI scenarios with *A* = 0.1 and *B* = 0.61, as shown with the dashed lines in Figure 9c,f.

## Conclusions

In summary, the potential of applying SAWs to modify the droplet impact dynamics on inclined surfaces is investigated in this paper. We have experimentally and numerically studied the effects of impact velocity, SAW direction and power, and surface inclination angle on the droplet impact behavior on a hydrophobic surface. Numerical results verified that SAWs could alter the energy budget of the impacting droplet and modify the impact dynamics. Applying the DSAW to the surface during the impingement process increases the kinetic energy of the droplet, leading to a faster detachment from the surface. On the other hand, by applying the USAW, the energy dissipation within the liquid medium is increased compared to those for the DSAW and FI scenarios. The slightly increased kinetic energy causes a faster detachment from the surface. The effects of SAW directions, substrate inclination angle, and impact velocity on the hydrodynamics of the droplet were examined and discussed in terms of droplet impact regime, contact time, maximum spreading, rebouncing angle, and droplet movement on the surface during the impact. Applying the SAWs, regardless of its direction, can avoid the deposition of the droplet on the inclined surface after the impact. This result shows the great potential of the SAW for applications in smart water-repellent surfaces.

Moreover, droplet contact time can be modified and controlled in a certain range by changing the power and direction of the propagating SAWs on the solid surface. Contact time control (not only reduction) is important for applications, such as spray cooling of reactors and electronic components. The presented simulation and experimental results show that using the SAWs, the contact time of the droplet on the inclined surfaces can be actively modified in a wide range. Additionally, the droplet rebouncing angles are varied by changing the SAW power and direction. Directing the droplet toward a certain target after impact onto an inclined surface could be useful in microfluidic applications such as 3D bioprinting. Our experimental results show that the rebouncing angle of the droplet can be modified effectively for different impact situations. Thus, the results illustrate the significant effect of acoustic waves on droplet impact on inclined surfaces. Therefore, we expect that SAW technology can be used in many applications such as smart self-cleaning, anti-icing, and anti-infection surfaces.

## Supporting Information Available

Effect of inclination angle and SAW direction on the interaction between gravitational and SAW forces; schematic views of the effects of SAW direction and inclination angle on the interaction of SAW and gravitational forces; ; schematic view of the SAW device; schematic illustration of the experimental setup; SEM image of the ZnO/Si SAW device surface; uncertainty analysis for droplet initial diameter *D*_0_ and droplet impact velocity *U*_0_ for different experimental cases as a function of droplet release height, *H*; explanation of the mathematical modeling; developed contact angle modeling; and numerical setup;input parameters and modeling setup for numerical simulation; explanation of the model validation; comparisons between experimental and simulation results of the droplet interface during the impact on a surface with an inclination angle of 30° for FI scenario, USAW scenario, and DSAW scenario; analysis of the dynamic contact angle; droplet normalized contact width versus time; temporal evolution of the apparent dynamic contact angle of leading and tailing edges for FI scenario, USAW scenario, and DSAW scenario; different impact regimes; and repeatability of the droplet rebouncing angle from the surface (PDF)
Droplet FI on a smooth hydrophobic surface with an inclination angle of 30° with *We* = 50; droplet impact in the presence of USAW with the power of 15 W (applied to the IDTs) on a smooth hydrophobic surface with an inclination angle of 30° with *We* = 50; droplet impact in the presence of DSAW with the power of 15 W (applied to the IDTs) on a smooth hydrophobic surface with an inclination angle of 30° with *We* = 50; droplet FI on a smooth hydrophobic surface with an inclination angle of 15° with *We* = 30; droplet impact in the presence of USAW with the power of 15 W (applied to the IDTs) on a smooth hydrophobic surface with an inclination angle of 15° with *We* = 30; and droplet impact in the presence of DSAW with the power of 15 W (applied to the IDTs) on a smooth hydrophobic surface with an inclination angle of 15° with *We* = 30 (ZIP)

## Abbreviationspara

SAW: surface acoustic wave
DSAW: downward surface acoustic wave
USAW: upward surface acoustic wave
FI: free impact
TPCL: three-phase contact line
CLSVOF: coupled level set volume of fluid
IDT: interdigital transducer
CFD: computational fluid dynamics
