# Supplementary material for: Dynamic Behavior of Droplet Impact on Inclined Surfaces with Acoustic Waves
Source: Langmuir. 2020 Aug 7;36(34):10175–86. doi: 10.1021/acs.langmuir.0c01628 (PMC8010791; doi:10.1021/acs.langmuir.0c01628)
Supplement: Supplementary file 1 — la0c01628_si_001.pdf [file la0c01628_si_001.pdf]

Supplementary material

Dynamic behavior of droplet impact on inclined surfaces with acoustic waves

Mehdi H. Biroun[1], Mohammad Rahmati[1], Ran Tao[1,2], Hamdi Torun[1], Mehdi Jangi[3], Yongqing Fu[1,]*

1: Faculty of Engineering and Environment, Northumbria University, Newcastle upon Tyne, NE1 8ST, UK

2: Shenzhen Key Laboratory of Advanced Thin Films and Applications, College of Physics and Optoelectronic Engineering, Shenzhen University 518060, China

3: Department of Mechanical Engineering, University of Birmingham, Birmingham, B15 2TT, UK

S1. Effect of inclination angle and SAW direction on the interaction between gravitational and SAW forces

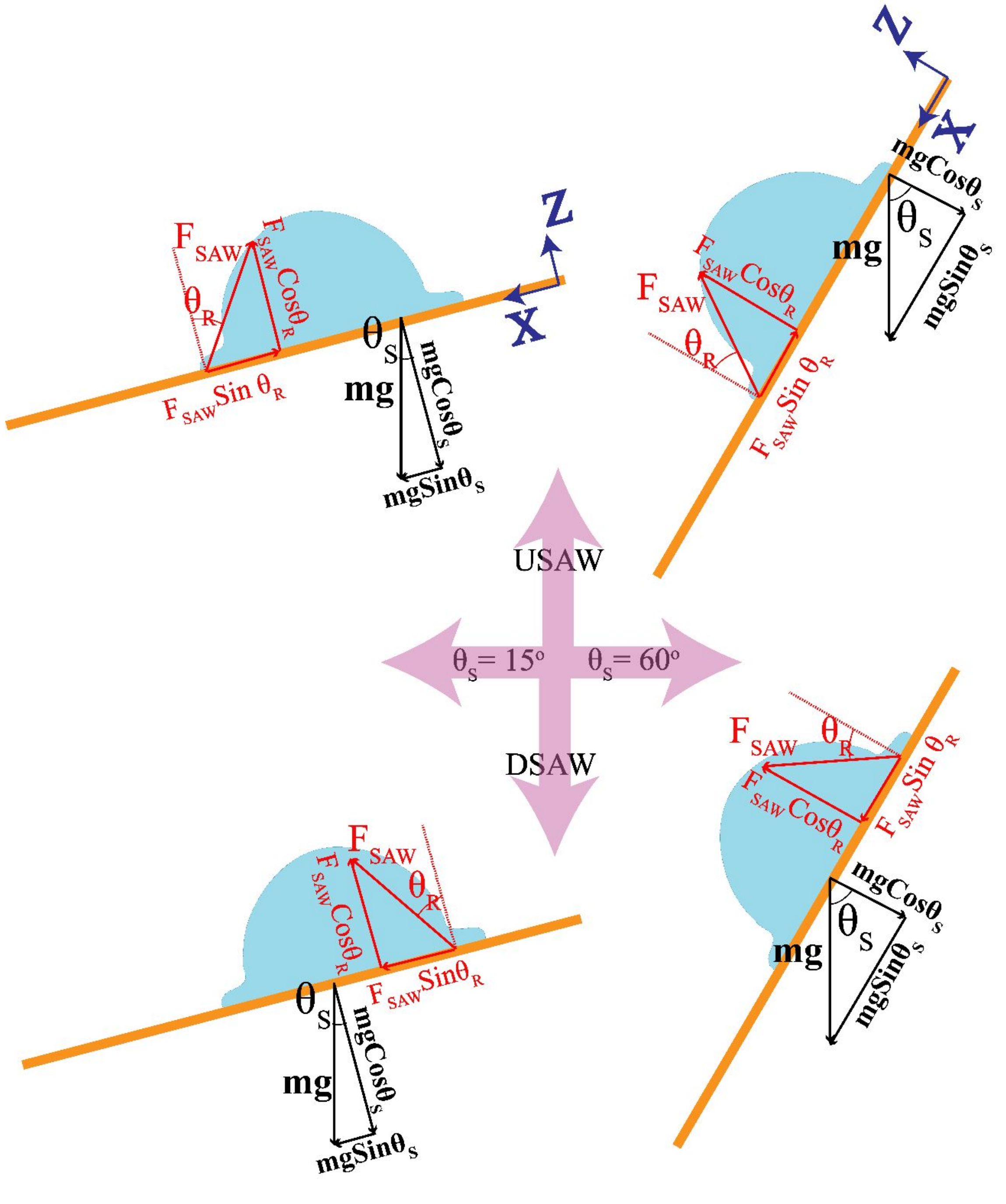

Figure S1. Schematic views of the effects of SAW direction and inclination angle on the interaction of SAW and gravitational forces.

S2. Experimental setup

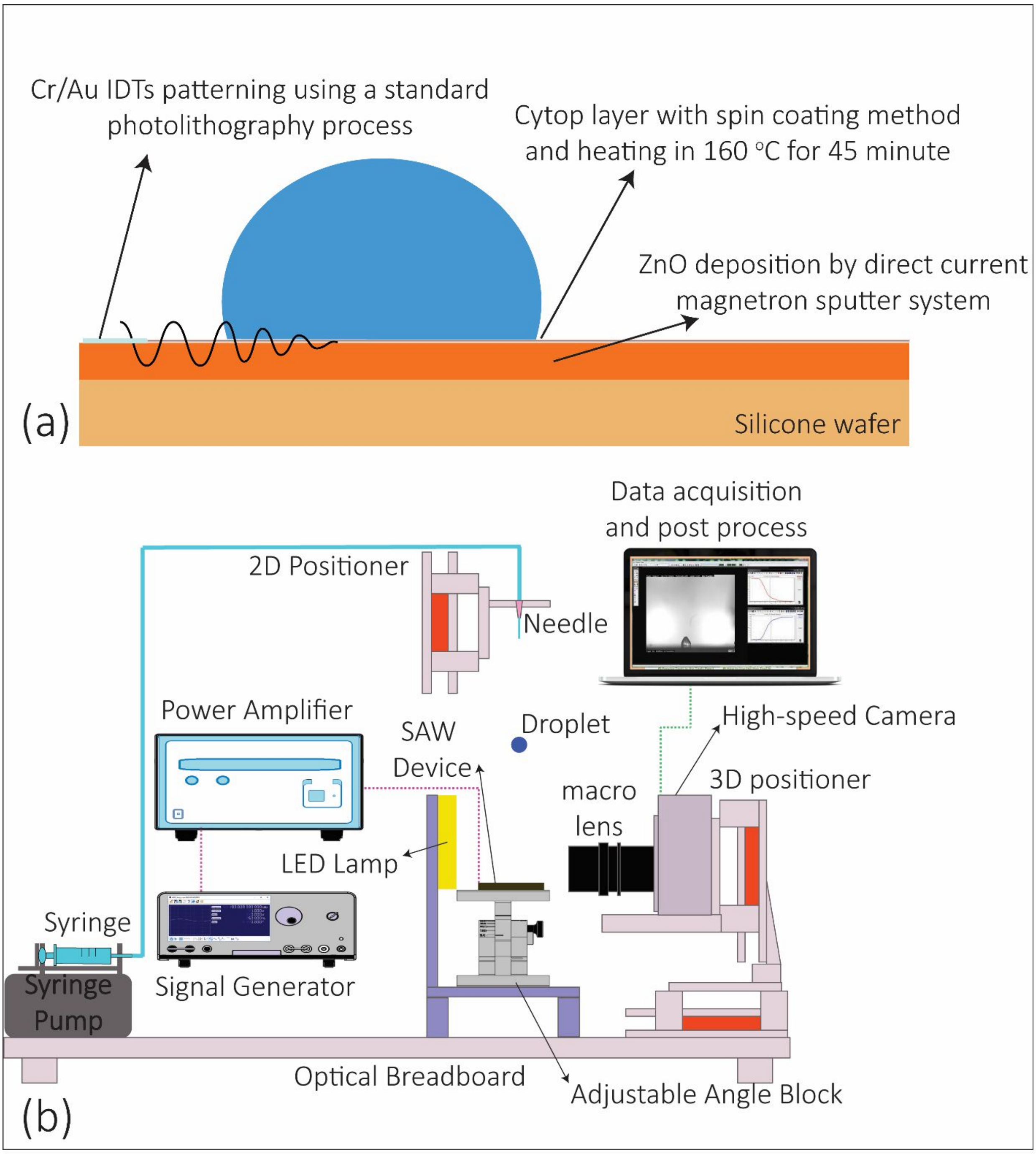

Figure S2. (a) Schematic view of the SAW device, (b) Schematic illustration of the experimental setup

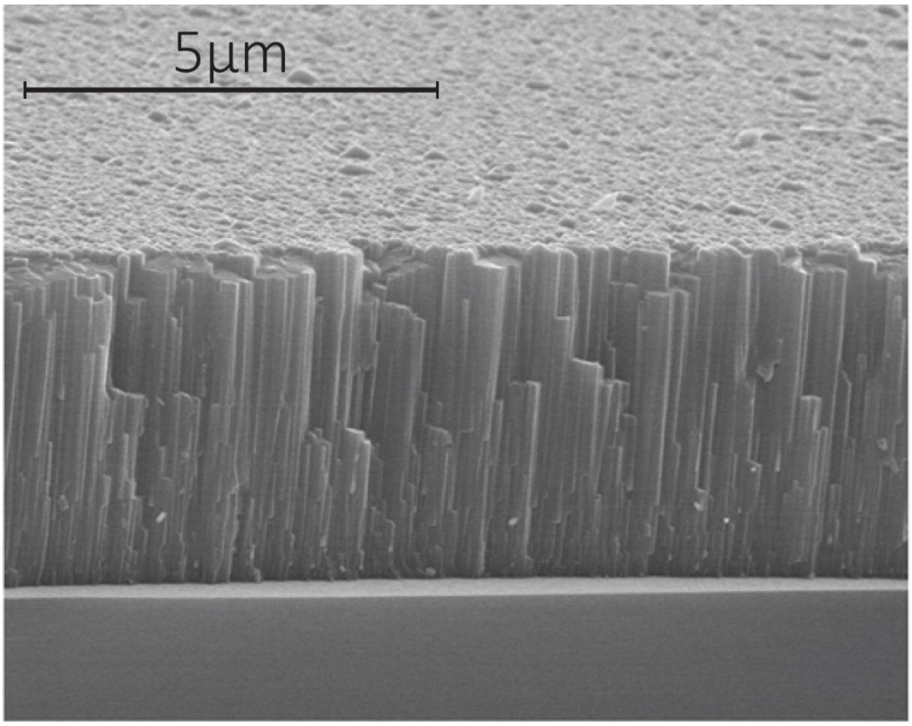

Figure S3. Scanning electron microscopy (SEM) image of the ZnO/Si SAW device surface.

S3. Uncertainty analysis

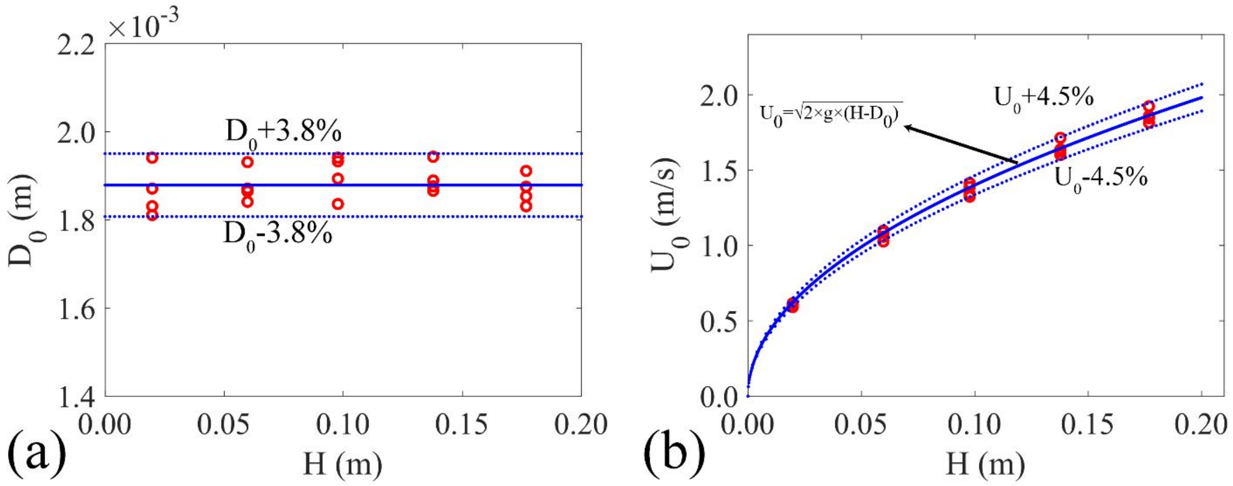

Figure S4. Uncertainty analysis for (a) Droplet initial diameter D_o; and (b) droplet impact velocity U_o for different experimental cases as a function of droplet release height, H.

S4. Mathematical modeling

The mathematical model for the two-phase flow was developed based on the CLSVOF method [1]. We assumed that the droplet and the solid surface have an equal temperature, and evaporation during impact is negligible. The conservation of momentum equation is then solved throughout the domain [1].

$$\frac{\partial}{\partial t}(\rho \boldsymbol{U}) + \nabla.(\rho \boldsymbol{U} \otimes \boldsymbol{U}) = -\nabla p + \nabla.\left[\mu(\nabla \boldsymbol{U} + \nabla^T \boldsymbol{U})\right] + \rho \boldsymbol{g} + \boldsymbol{f_\sigma} + \boldsymbol{f_{SAW}} \qquad \text{(s1)}$$

where symbols in bold are used for vector and tensor variables. $\boldsymbol{U}$ is velocity, $\boldsymbol{g}$ is the gravitational acceleration vector, p is pressure, ρ and μ are density and dynamic viscosity respectively, $\boldsymbol{f_\sigma}$ is the surface tension force, and $\boldsymbol{f_{SAW}}$ is the SAW force.

In the volume of fluid (VOF) method [2], both continuous fields (liquid) and discrete (gas) fields are defined by the volume fraction α which lies between 0 and 1. The volume fraction follows the conservation law:

$$\frac{\partial \alpha}{\partial t} + \nabla.(\boldsymbol{U}\alpha) = 0 \qquad \text{(s2)}$$

The volume fraction is defined as:

$$\alpha = \begin{cases} 0 & Gas\ Phase \\ 1 & Liquid\ Phase \\ 0 < \alpha < 1 & Interface \end{cases} \qquad \text{(s3)}$$

Density and viscosity in each cell are expressed by the volume fraction:

$$\rho = \rho_l \alpha + \rho_g (1 - \alpha) \qquad \text{(s4)}$$

$$\mu = \mu_l \alpha + \mu_g (1 - \alpha) \qquad \text{(s5)}$$

where indices "l " and "g" indicate liquid and gas, respectively. The volumetric surface tension force, f_σ, can be calculated by a method proposed by Brackbill et al. [3]

$$\boldsymbol{f_\sigma} = \gamma_{LV} \kappa(\alpha) \nabla \alpha \qquad \text{(s6)}$$

where γ_{LV} is surface tension coefficient and $\kappa(\alpha)$ is the curvature of the interface. The curvature is the magnitude of the interface normal flux at each cell, and it indicates the direction of this flux.

$$\kappa(\alpha) = -\nabla.\frac{\nabla \alpha}{|\nabla \alpha|} \qquad \text{(s7)}$$

The precise calculation of the curvature at the interface is challenging since α is a discontinuous step function at the interface [1,4]. To obtain a smooth and sharp interface and also conserve the mass within the interface, we

coupled the level-set and VOF method using the coupling method proposed by Sussman and Puckett [1]. In this method, the LS field, ϕ, is defined as:

$$\phi = \begin{cases} < 0 & Gas\ Phase \\ > 0 & Liquid\ Phase \\ 0 & Interface \end{cases} \tag{s8}$$

At each time step, it is assumed that the interface position is at $\alpha = 0$ and ϕ is initialized as:

$$\phi_0 = (2\alpha - 1).\Gamma \tag{s9}$$

where $\Gamma = 0.75\Delta x$, is a non-dimensional number which is a function of the mesh size, Δx. Re-initialization equation is then solved to redistance the ϕ_0 value:

$$\frac{\partial \phi}{\partial \tau} = S(\phi_0)(1 - |\nabla \phi|) \tag{s10}$$

where $S(\phi_0)$ is the sign function of initial LS function and τ the artificial time. The curvature at the sharper interface defined by LS function can be determined as:

$$\kappa(\phi) = -\nabla.\frac{\nabla \phi}{|\nabla \phi|} \tag{s11}$$

The volumetric surface tension force can be estimated using $\kappa(\phi)$ as:

$$\boldsymbol{f_\sigma} = \gamma_{LV}\kappa(\phi)\delta(\phi)\nabla\phi \tag{s12}$$

where

$$\delta(\phi) = \begin{cases} 0 |\phi| > \epsilon \\ \frac{1}{2\epsilon}\left(1 + cos\left(\frac{\pi\phi}{\epsilon}\right)\right)|\phi| \le \epsilon \end{cases} \tag{s13}$$

where ϵ is the interface thickness and is defined in a way that the two-phase interface does not smear beyond a specific number of the cells. In this model, $\epsilon = 0.75\Delta x$. The mixture's density and viscosity are then calculated by:

$$\rho(\phi) = \rho_g + (\rho_l - \rho_g)H(\phi) \tag{s14}$$

$$\mu(\phi) = \mu_g + (\mu_l - \mu_g)H(\phi) \tag{s15}$$

where

$$H(\phi) = \begin{cases} 0 & |\phi| < -\epsilon \\ \frac{1}{2}\left[1 + \frac{\phi}{\epsilon} + \frac{1}{\pi} sin\left(\frac{\pi\phi}{\epsilon}\right)\right] & |\phi| \leq \epsilon \\ 1 & |\phi| > \epsilon \end{cases} \quad \text{(s16)}$$

S5. Contact Angle Modelling

Instead of calculation of the contact angle as a part of the simulation, here we define the macroscopic contact angle as a wall boundary condition. The experimental movies showed that the three-phase contact line is moving along the solid surface, and the contact angle is limited to advancing, θ_{adv} and receding, θ_{rec} contact angles. To implement the wall boundary condition for the CLSVOF model, we developed a model based on the dynamic contact angle boundary condition for interFoam VOF solver in OpenFOAM. In this method, the surface normal vector of the LS and VOF functions of the wall cell in the interface are rotated in a way that they are equal to the desired contact angle. The dynamic contact angle is calculated first by defining an equation which is related to the contact angle to Capillary number, $Ca = {U_{cl}\mu_l}/{\gamma_{LV}}$. The contact line velocity, U_{cl}, is the component of the cell center velocity parallel to the wall and normal to the interface from liquid toward the gas phase. Various mathematical models have been proposed for the calculation of dynamic contact angles [5]. In this research, we applied the combined approaches proposed by Afkhami et al. [6] and bracke et al.[7] to calculate the dynamic contact angle, e.g.,

$$\cos \theta_d = \cos \theta_s - (\cos \theta_s + 1).\mathrm{K}_{DCA}.(\mathrm{Ca}.\ln{(2\mathrm{K}/(\Delta x))})^{0.54} \qquad \text{(s17)}$$

where θ_s is the static apparent contact angle, and $\mathrm{K}_{DCA} = 9.96$ and $k = 0.02$ are the model parameters. To limit the calculated contact angle from equation (s17) to advancing and receding contact angles, we use the approach proposed by Yokoi et al. [8]:

$$\theta_d = \begin{cases} \max[\theta_d, \theta_{adv}] \text{ if } U_{CL} < 0 \\ \min[\theta_d, \theta_{rec}] \text{ if } U_{CL} \geq 0 \end{cases} \qquad \text{(s18)}$$

S6. Numerical setup

For the numerical simulation, firstly, the droplet drop from a height of 10 cm down to 0.5 cm from the solid surface was simulated in the domain-A. Domain-A was an inclined rectangular box with a dimension of 105×5×5 mm and an inclination angle of 30 degree. Two-step local mesh refinement along the Z-direction was employed in the droplet movement area. Initially, a droplet with a radius of 0.938 mm and an initial velocity of 0 m/s was set to be 100 mm above the solid surface. The simulation was performed for 150 ms for the droplet drop at 25 ºC. The gravity vector was rotated with Θ= 30º in this simulation. Then the simulation results of the domain-A after 150 ms were mapped to the Domain-B. Based on the impact velocity, the maximum spreading diameter, and liquid properties, the geometry of the domain-B was decided to be a rectangular box with dimensions of 12×7×6 mm^3 (e.g., x-y-z directions).

To couple the velocity and pressure fields, PIMPLE algorithm of OpenFOAM was applied [9]. The time step was limited by a maximum Courant number of 0.3. To generate the mesh in simulations, the BlockMesh internal mesh generator of OpenFOAM was used [10].

To relax the “no-slip” boundary condition on the three-phase contact line, Navier slip boundary condition was implemented [6] and specified for the bottom boundary. In this method, the magnitude of the slip on the patch (z = 0) is proportional to the shear stress through a “slip length” coefficient, λ.

$$u|_{z=0} - U = \lambda \frac{\partial \boldsymbol{U}}{\partial x}|_{z=0} \quad \text{(s19)}$$

where u is the slip velocity, and U is the solid patch movement velocity. After a series of test simulations, the slip length was fixed at 0.001 for all the simulations in this study. The dynamic contact angle method explained in the previous section was implemented based on the DynamicContactAngle model of OpenFOAM[10]. This boundary condition was applied for the bottom patch of the domain for both LS and VOF functions. The input parameters, fluid, and SAW properties, boundary conditions, and solution methods are presented in Table SI.

To compare the simulation results with experimental results for model and boundary condition validation, we chose a power of 20 *w* for both USAW and DSAW scenarios. As seen in equation (2) in the main text, term *A* represents the applied SAW power. To fit the applied power in experimental results to *A*, here we use the following equation [11] and set the value of *A* to be 1452×10^{-10} m.

$$\frac{A}{\lambda} = 6.72 \times 10^{-6} \times P^{1.94} + 1.52 \times 10^{-6} \times P^{0.91} \quad \text{(s20)}$$

Table SI. Input parameters and modeling setup for numerical simulation

Input parameter	Value
Advancing contact angle	123º
Receding contact angle	95º
Static contact angle	104º
Surface tension coefficient	0.0707106 Kgs^{-2}
Wave amplitude	1452 Å
SAW frequency	66.05 MHz
Wavenumber [4]	2700 m^{-1}
Wave attenuation coefficient [4]	2.47

S7. Model validation

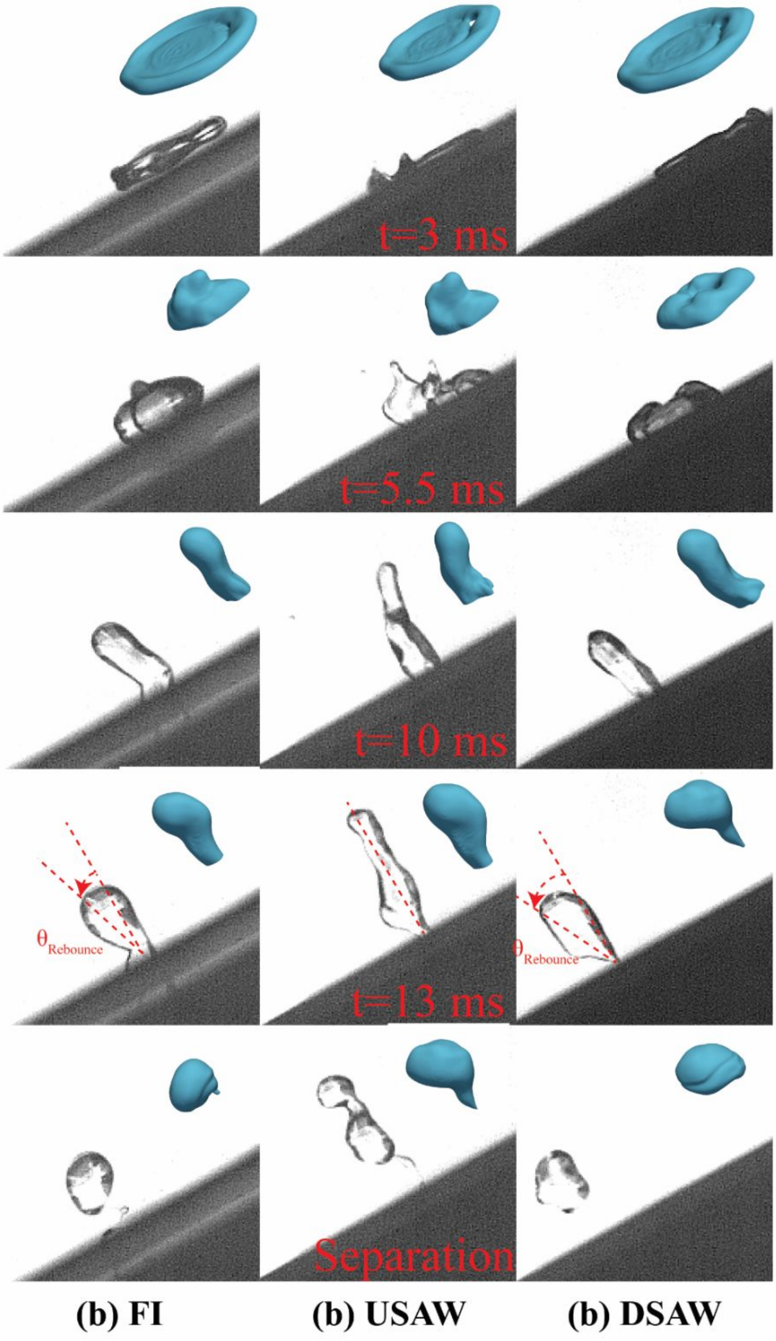

Figure S5. Comparisons between experimental and simulation results of the droplet interface during the impact on a surface with an inclination angle of 30° for (a) FI scenario, (b) USAW scenario, and (c) DSAW scenario. In both simulation and experiments, a droplet with a volume of 3.5 µl and We number of 50 is impacting on the surface.

S8. Analysis of dynamic contact angle

The temporal evolution of the contact width during the impact of droplet for the designed scenarios is presented in Figure S6(a). The contact line retracts after reaching its maximum spreading diameter ($\beta \cong 2.2$). Then, the three-phase contact line (TPCL) starts to retract towards the center of the droplet until the contact width ratio is around 0.7. This phenomenon happens in the first 10 ms of the impact. Near the end of the retract phase, since the droplet does not have enough energy to be fully separated from the surface, the contact width ratio stays nearly a constant (between 12-15 ms after the onset of the impact). During this period, the TPCL is pinned to the surface, and only the interface between the droplet and solid surface is vibrating (see supplementary video V4). After 16 ms, due to the conversion of surface energy into kinetic energy and also the component of the gravitational force along the surface, the leading-edge of the droplet moves downward on the surface. In contrast, the tailing-edge of the impacting droplet is almost stationary, which leads to another spreading phase of the droplet. The spreading lasts until the contact width ratio reaches a value of 1.4, which then remains a nearly constant value.

The apparent dynamic contact angle (ADCA) of the droplet as a function of time for the FI scenario is presented in Figure S6(b). The complex interactions among the resistive, viscous, surface tension and gravitational forces result in different contact angles. Also, since the impact happens on an inclined surface, the contact line motion (and thus contact angle dynamics) is a function of TPCL motion during the impingement as well as droplet motion along the X-direction (induced by the gravitational force). The ADCA values of both leading and tailing-edges of the droplet during most of the spreading phase show small variations, which is in a good agreement with literature [2]. The ADCA at the tailing-edge starts to decrease as the retracting period commences and then stays almost a constant during the rest of the retract phase. The ADCA at the leading edge roughly follows a similar pattern, but with a delay of ~1 ms for the first period of 6 ms. Between 11-20 ms, the tailing-edge contact angle still shows the small variations.

On the other hand, the leading-edge ADCA has increased sharply up to 140° between ~11-15 ms and then decreased to 100° afterward. Another cycle of decrease/increase of the ADCA is observed between the periods of ~18-22 ms. Apparently, these repeated cycles in leading-edge ADCA is due to the droplet wobbling as a result of the interactions between inertia and capillary forces.

By applying the SAW to the droplet, in the area directly affected by the SAW energy, the ADCA tends to shows small variations, which is mainly due to the domination of the SAW force in that area. For instance, the ADCA of the leading-edge of the droplet effected by USAW shows minor variations during the droplet impact, except between 4-6 ms in which the interface wobbling due to inertia changes the ADCA for a few milliseconds. During the separation phase, the droplet is detached from the surface along the Rayleigh angle. For the DSAW scenario, since the gravitational force component and SAW forces have the same direction along the X-direction, the interface wobbling during the interface is not as significant as that for the USAW scenario. Thus, the ADCA for both tailing and leading edges only show minor variations (see figure S6(d)).

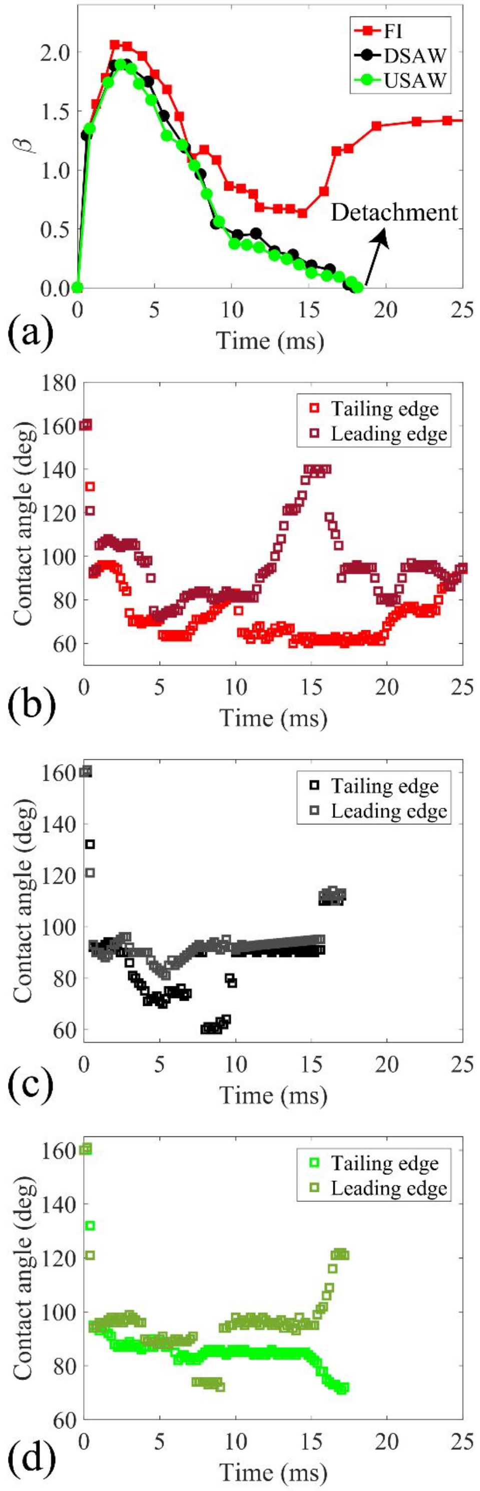

Figure S6. (a) Droplet normalized contact width versus time. Lines are provided as a guide for the eyes. Temporal evolution of apparent dynamic contact angle of leading and tailing edges for (b) FI scenario, (c) USAW scenario, (d) DSAW scenario.

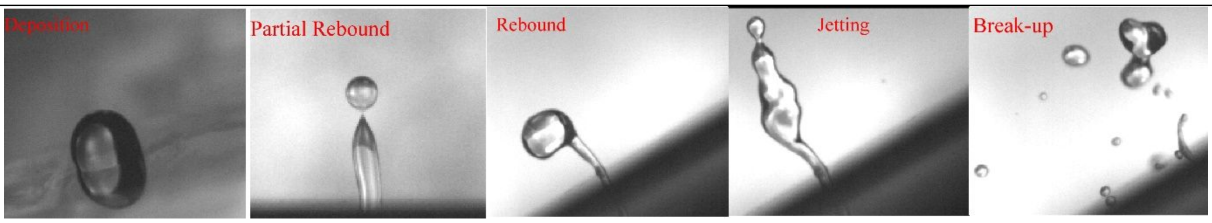

Figure S7. obtaining diverse impact regimes on the inclined surfaces by applying SAW

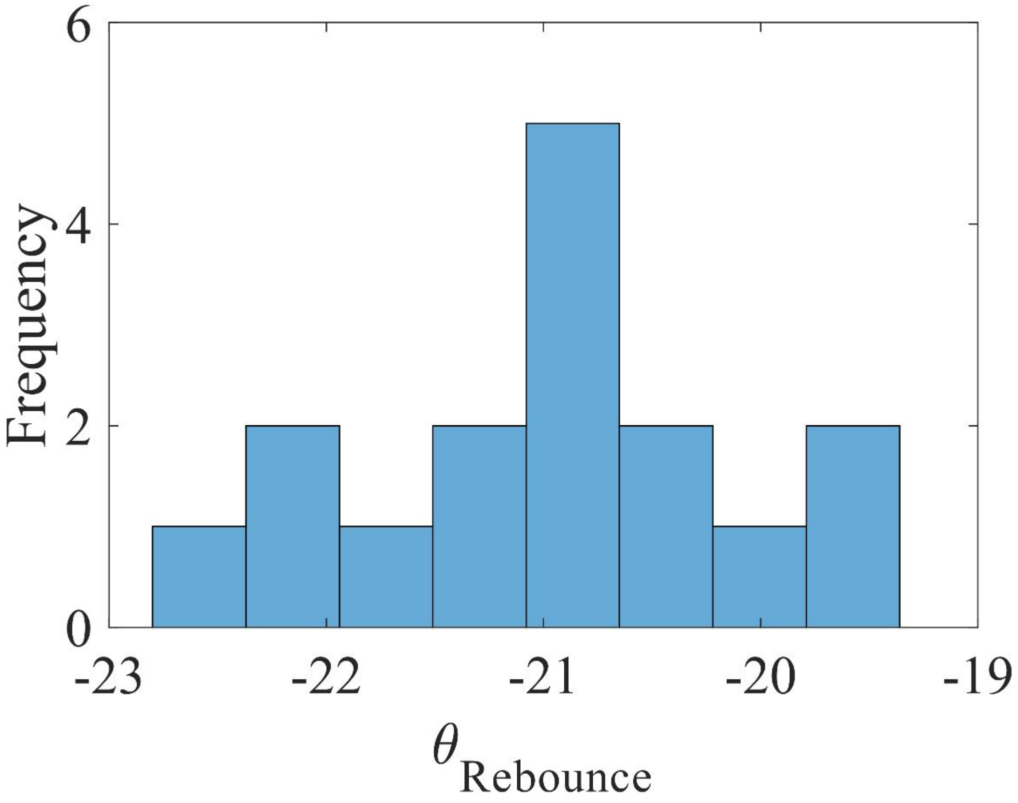

Figure S8. Frequency of droplet rebouncing angle for 16 experiments on a surface with an inclination angle of 15° and droplet impact velocity of 1.4 m/s. To test the repeatability of the droplet rebouncing angle, the experiments war repeated four times a day in 5 consecutive days. During the impact, USAW with the power of 35 w was propagating on the surface.

REFRENCES

(1) Sussman, M.; Puckett, E. G. A Coupled Level Set and Volume-of-Fluid Method for Computing 3D and Axisymmetric Incompressible Two-Phase Flows. *J. Comput. Phys.* **2000**, *162* (2), 301–337. https://doi.org/10.1006/jcph.2000.6537.

(2) Hirt, C. W.; Nichols, B. D. Volume of Fluid (VOF) Method for the Dynamics of Free Boundaries. *J. Comput. Phys.* **1981**, *39* (1), 201–225. https://doi.org/10.1016/0021-9991(81)90145-5.

(3) Brackbill, J. U.; Kothe, D. B.; Zemach, C. A Continuum Method for Modeling Surface Tension. *J. Comput. Phys.* **1992**, *100* (2), 335–354. https://doi.org/10.1016/0021-9991(92)90240-Y.

(4) Jangi, M.; Luo, J. T.; Tao, R.; Reboud, J.; Wilson, R.; Cooper, J. M.; Gibson, D.; Fu, Y. Q. Concentrated Vertical Jetting Mechanism for Isotropically Focused Zno/Si Surface Acoustic Waves. *Int. J. Multiph. Flow* **2019**, *114*, 1–8. https://doi.org/10.1016/j.ijmultiphaseflow.2019.02.002.

(5) Malgarinos, I.; Nikolopoulos, N.; Marengo, M.; Antonini, C.; Gavaises, M. VOF Simulations of the Contact Angle Dynamics during the Drop Spreading: Standard Models and a New Wetting Force Model. *Adv. Colloid Interface Sci.* **2014**, *212*, 1–20. https://doi.org/10.1016/j.cis.2014.07.004.

(6) Afkhami, S.; Zaleski, S.; Bussmann, M. A Mesh-Dependent Model for Applying Dynamic Contact Angles to VOF Simulations. *J. Comput. Phys.* **2009**, *228* (15), 5370–5389. https://doi.org/10.1016/j.jcp.2009.04.027.

(7) Fries, N.; Dreyer, M. The Transition from Inertial to Viscous Flow in Capillary Rise. *J. Colloid Interface Sci.* **2008**, *327* (1), 125–128. https://doi.org/10.1016/j.jcis.2008.08.018.

(8) Yokoi, K.; Vadillo, D.; Hinch, J.; Hutchings, I. Numerical Studies of the Influence of the Dynamic Contact Angle on a Droplet Impacting on a Dry Surface. *Phys. Fluids* **2009**, *21* (7), 1–12. https://doi.org/10.1063/1.3158468.

(9) Dianat, M.; Skarysz, M.; Hodgson, G.; Garmory, A.; Passmore, M. Coupled Level-Set Volume of Fluid Simulations of Water Flowing Over a Simplified Drainage Channel With and Without Air Coflow. *SAE Int. J. Passeng. Cars - Mech. Syst.* **2017**, *10* (1), 2017-01–1552. https://doi.org/10.4271/2017-01-1552.

(10) Milas, M.; Yu, D.; Lang, A.; Ge, T.; Feig, B.; El-Naggar, A. K.; Pollock, R. E. Adenovirus-Mediated P53 Gene Therapy Inhibits Human Sarcoma Tumorigenicity. *Cancer Gene Ther.* **2000**, *7* (3), 422–429. https://doi.org/10.1038/sj.cgt.7700141.

(11) Biroun, M. H.; Rahmati, M. T.; Jangi, M.; Tao, R.; Chen, B. X.; Fu, Y. Q. Computational and Experimental Analysis of Droplet Transportation/Jetting Behaviours Driven by Thin Film Surface Acoustic Waves. *Sensors Actuators, A Phys.* **2019**, *299*, 111624. https://doi.org/10.1016/j.sna.2019.111624.

(12) Vadillo, D. C.; Soucemarianadin, A.; Delattre, C.; Roux, D. C. D. Dynamic Contact Angle Effects onto the Maximum Drop Impact Spreading on Solid Surfaces. *Phys. Fluids* **2009**, *21* (12), 1–8. https://doi.org/10.1063/1.3276259.
