# Supplementary figures and images for: Dynamic Behavior of Droplet Impact on Inclined Surfaces with Acoustic Waves
Source: Langmuir. 2020 Aug 7;36(34):10175–86. doi: 10.1021/acs.langmuir.0c01628 (PMC8010791; doi:10.1021/acs.langmuir.0c01628)

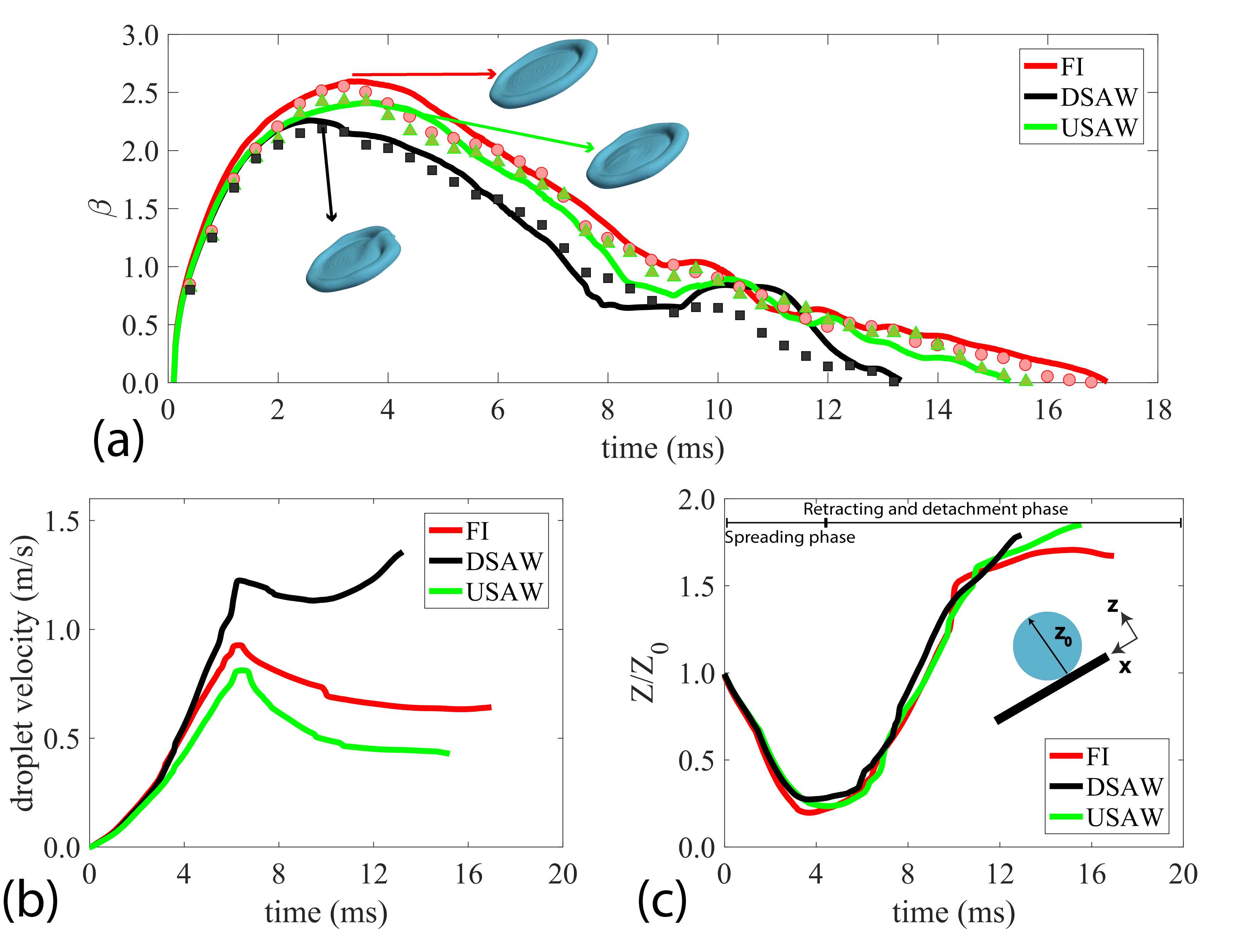

Supplement: Supplementary file 2 — la0c01628_si_002.zip [file la0c01628_si_002.zip › Graphics/Figure 2.tif]

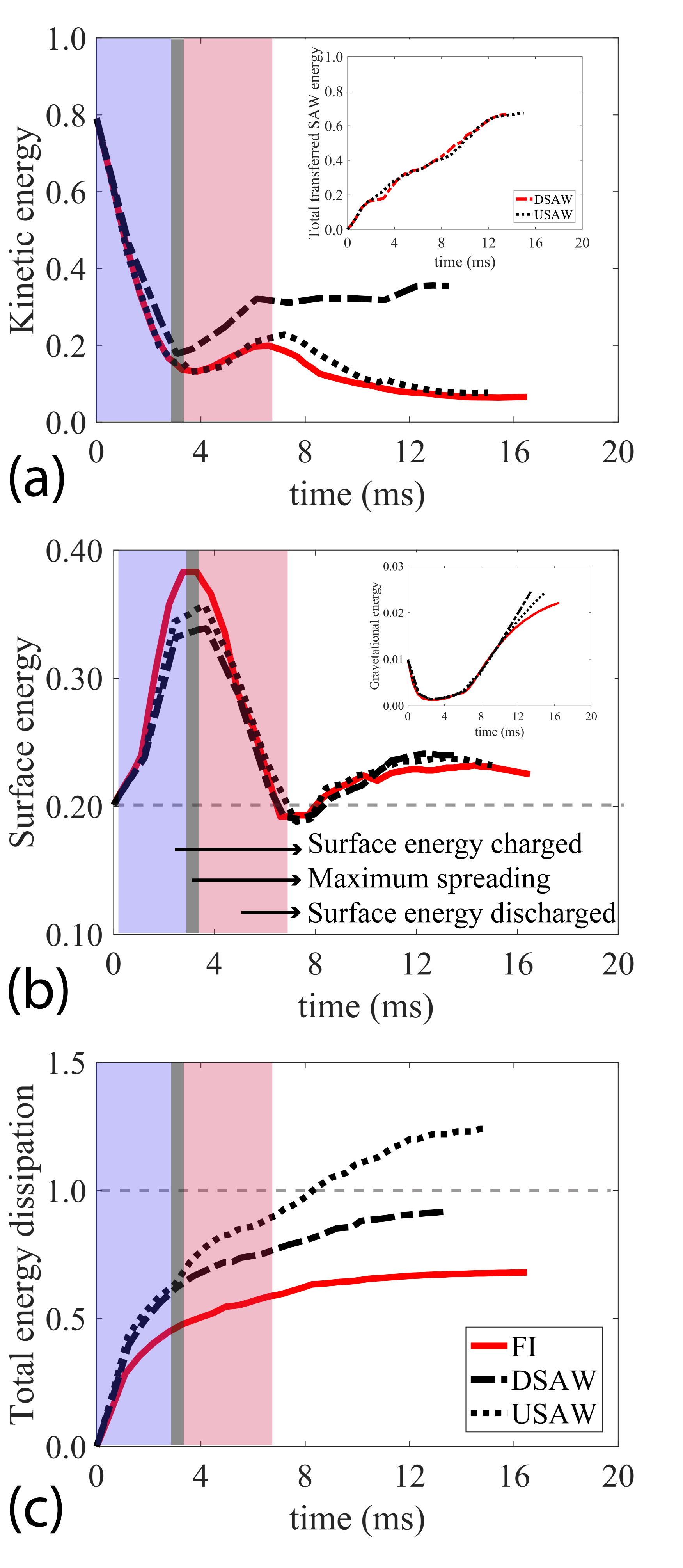

Supplement: Supplementary file 2 — la0c01628_si_002.zip [file la0c01628_si_002.zip › Graphics/Figure 4.tif]

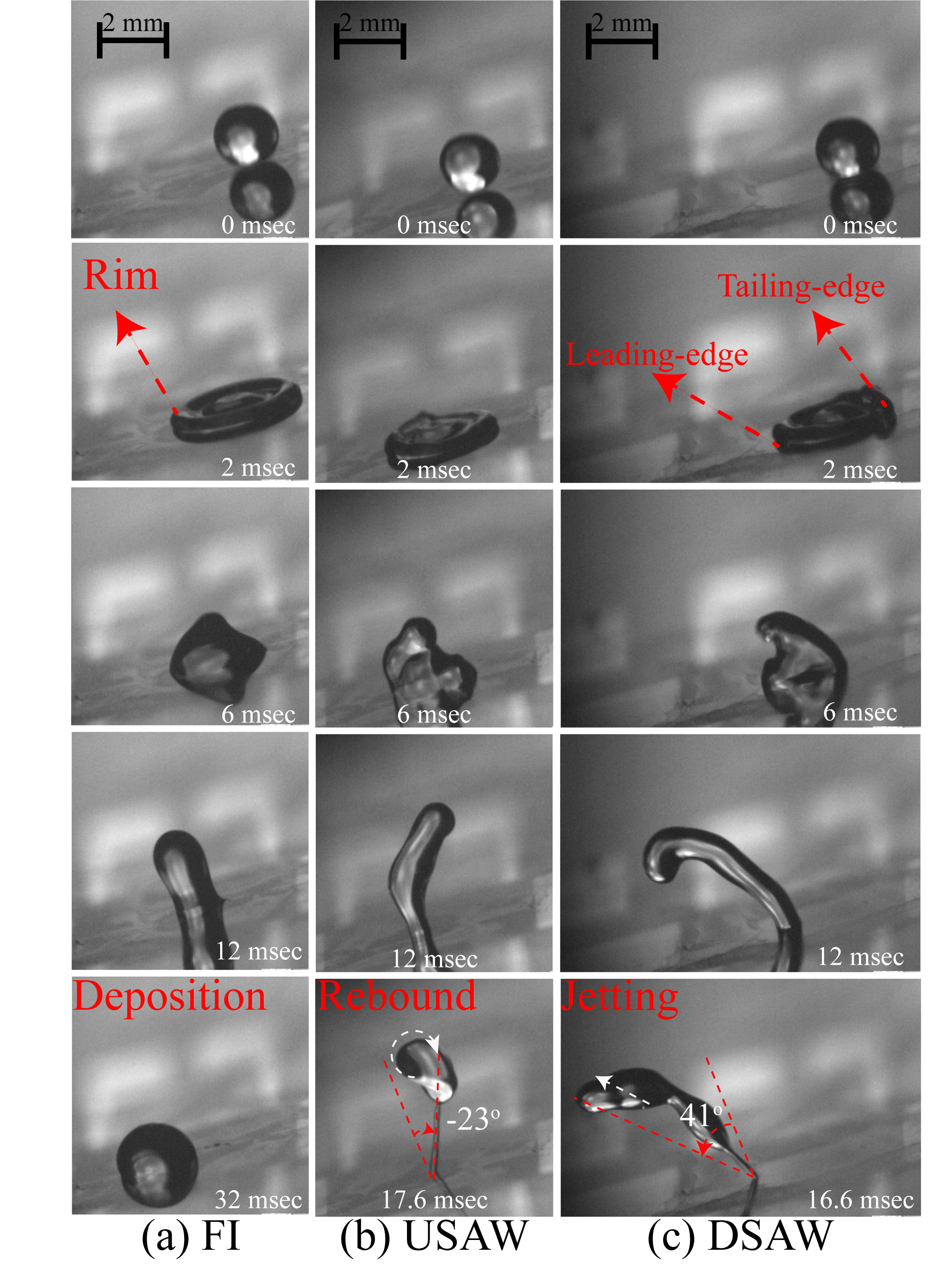

Supplement: Supplementary file 2 — la0c01628_si_002.zip [file la0c01628_si_002.zip › Graphics/Figure 5.tif]

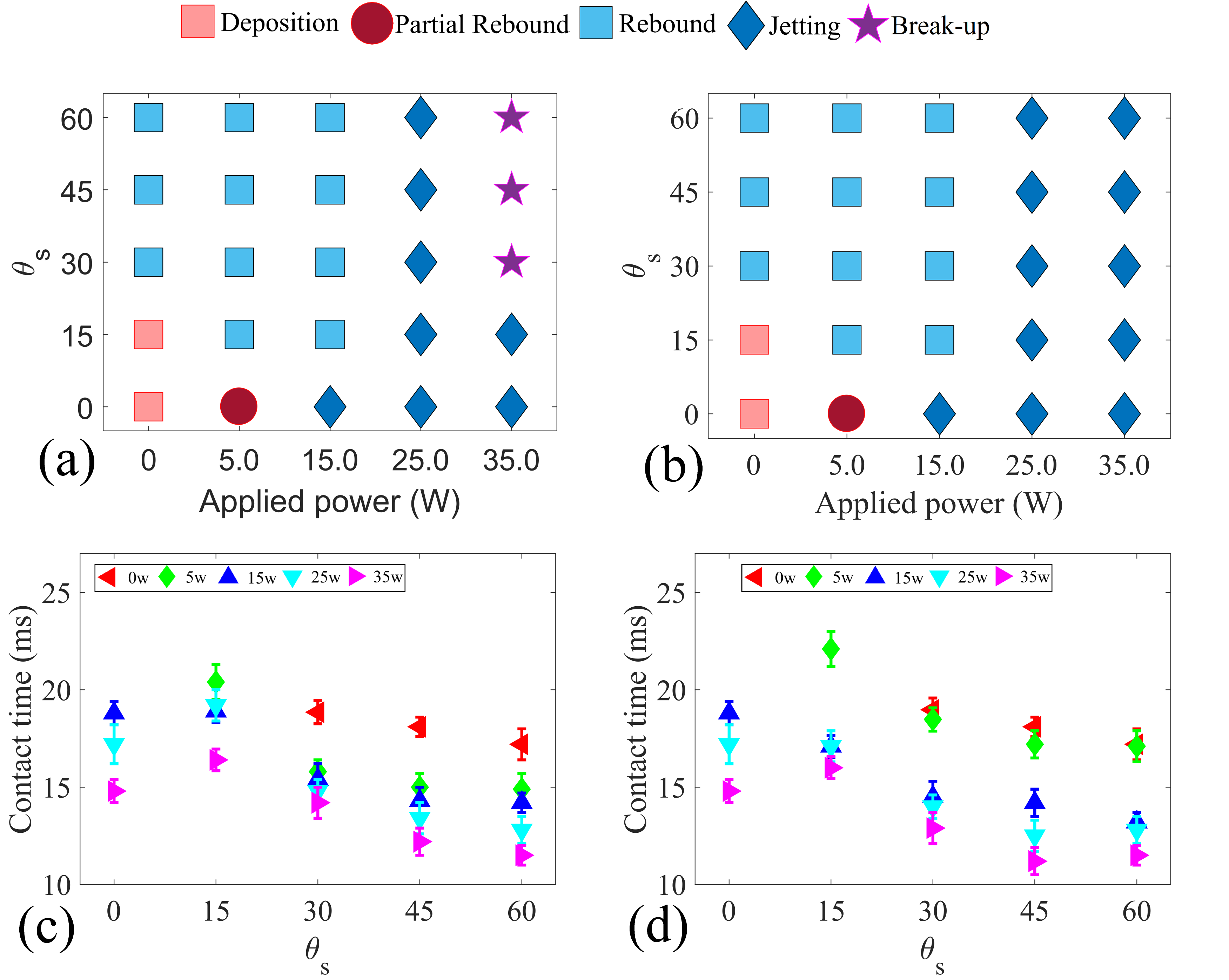

Supplement: Supplementary file 2 — la0c01628_si_002.zip [file la0c01628_si_002.zip › Graphics/Figure 6.tif]

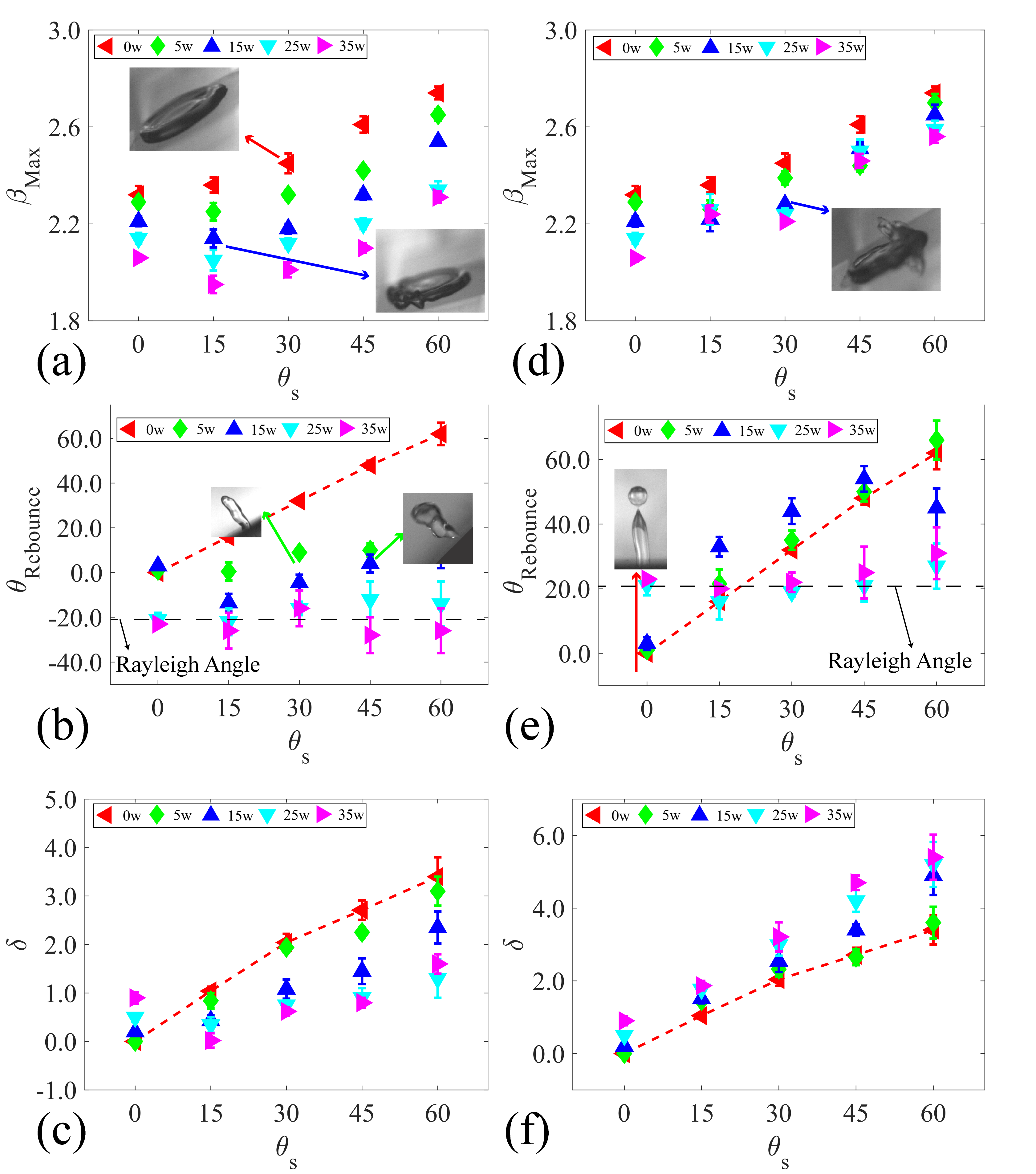

Supplement: Supplementary file 2 — la0c01628_si_002.zip [file la0c01628_si_002.zip › Graphics/figure 7.tif]

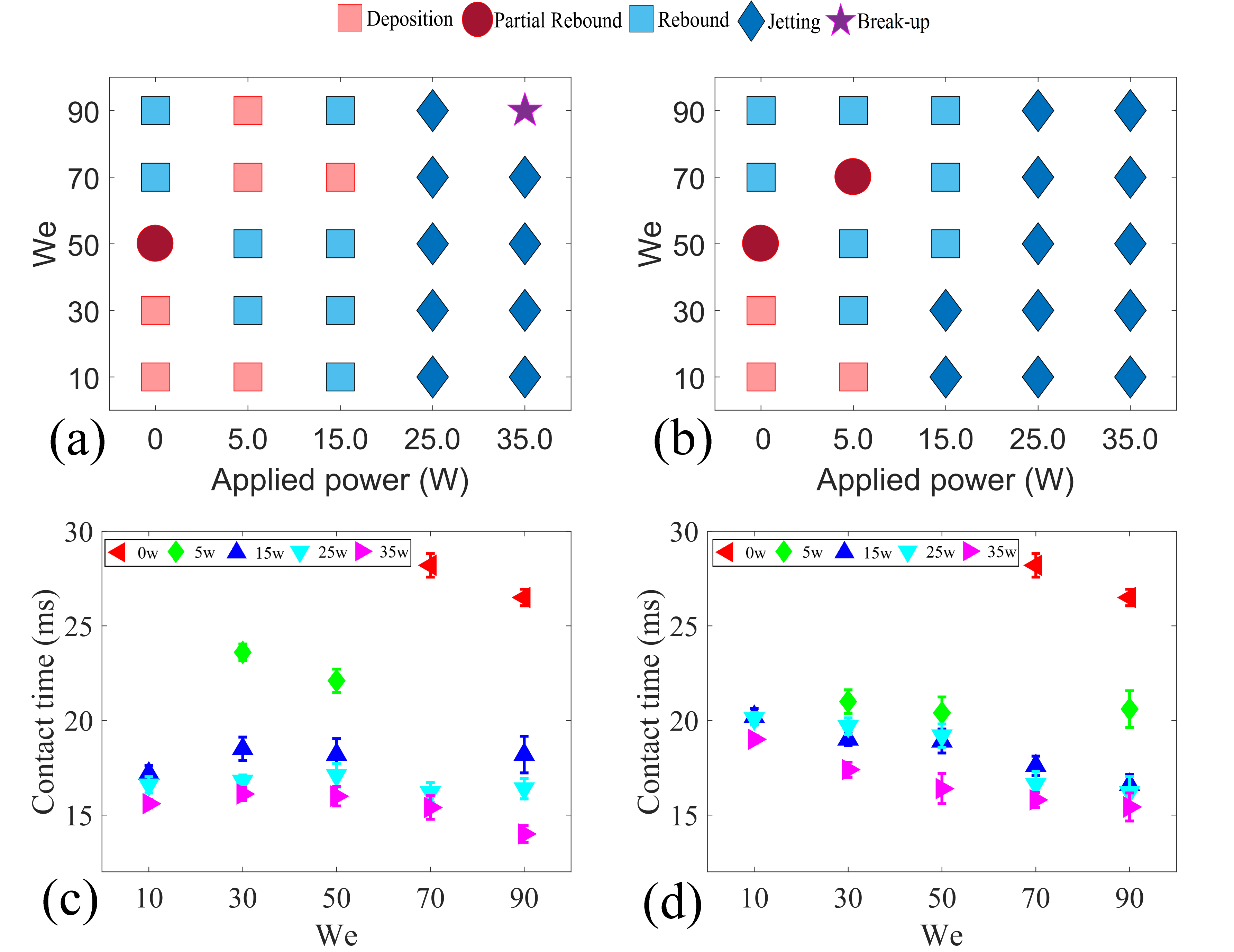

Supplement: Supplementary file 2 — la0c01628_si_002.zip [file la0c01628_si_002.zip › Graphics/Figure 8.tif]

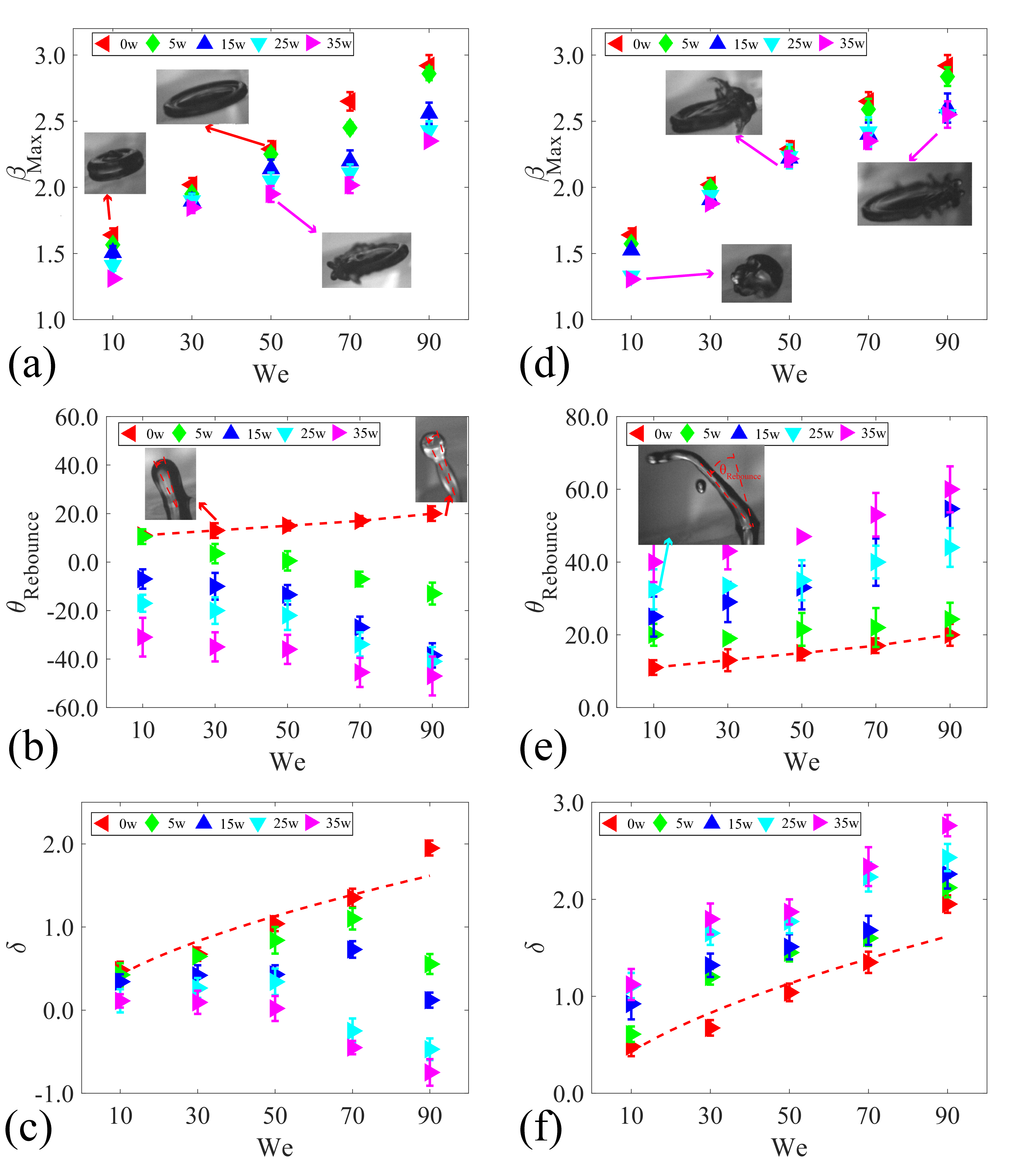

Supplement: Supplementary file 2 — la0c01628_si_002.zip [file la0c01628_si_002.zip › Graphics/Figure 9.tif]

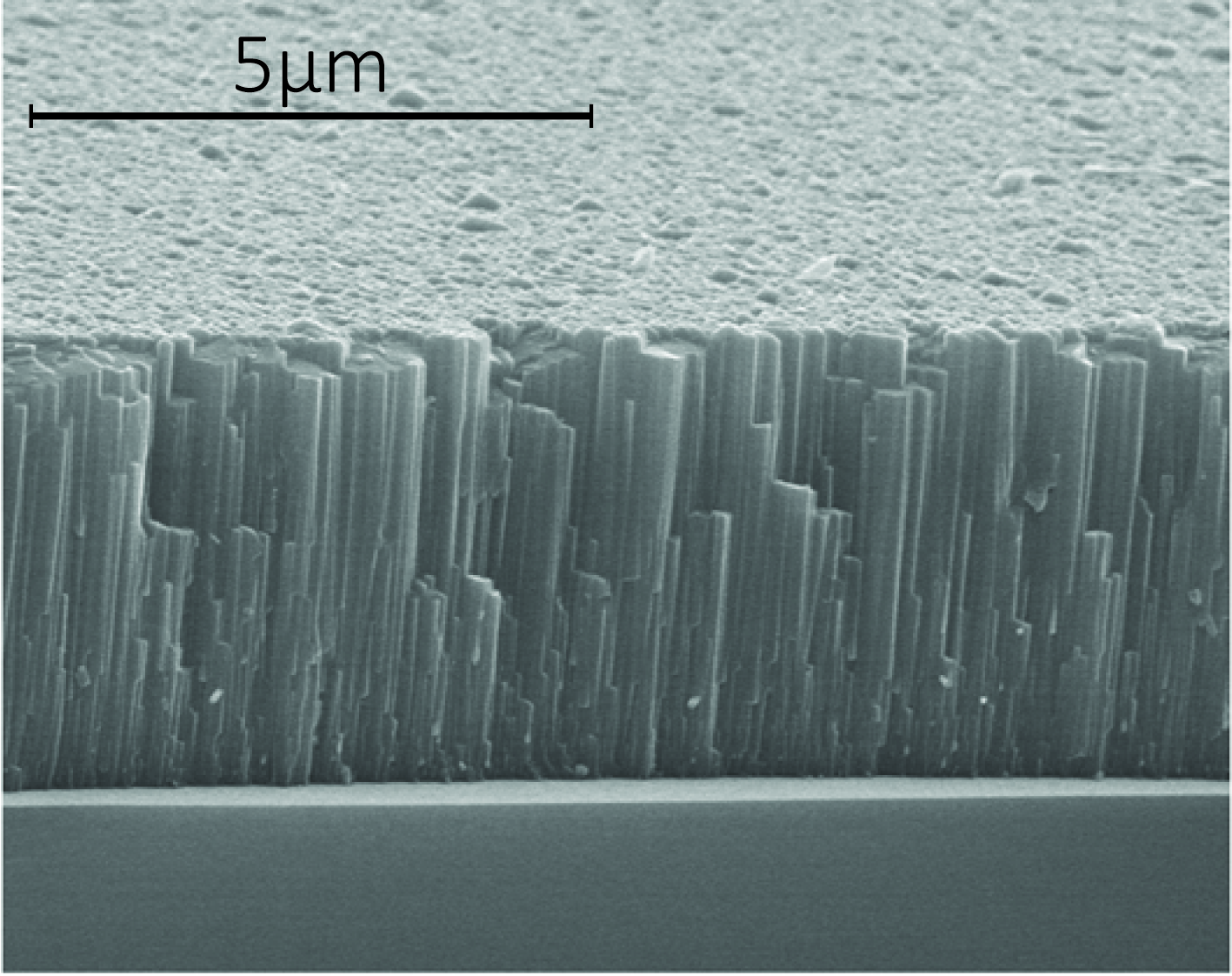

Supplement: Supplementary file 2 — la0c01628_si_002.zip [file la0c01628_si_002.zip › Graphics/Figure S3.tif]

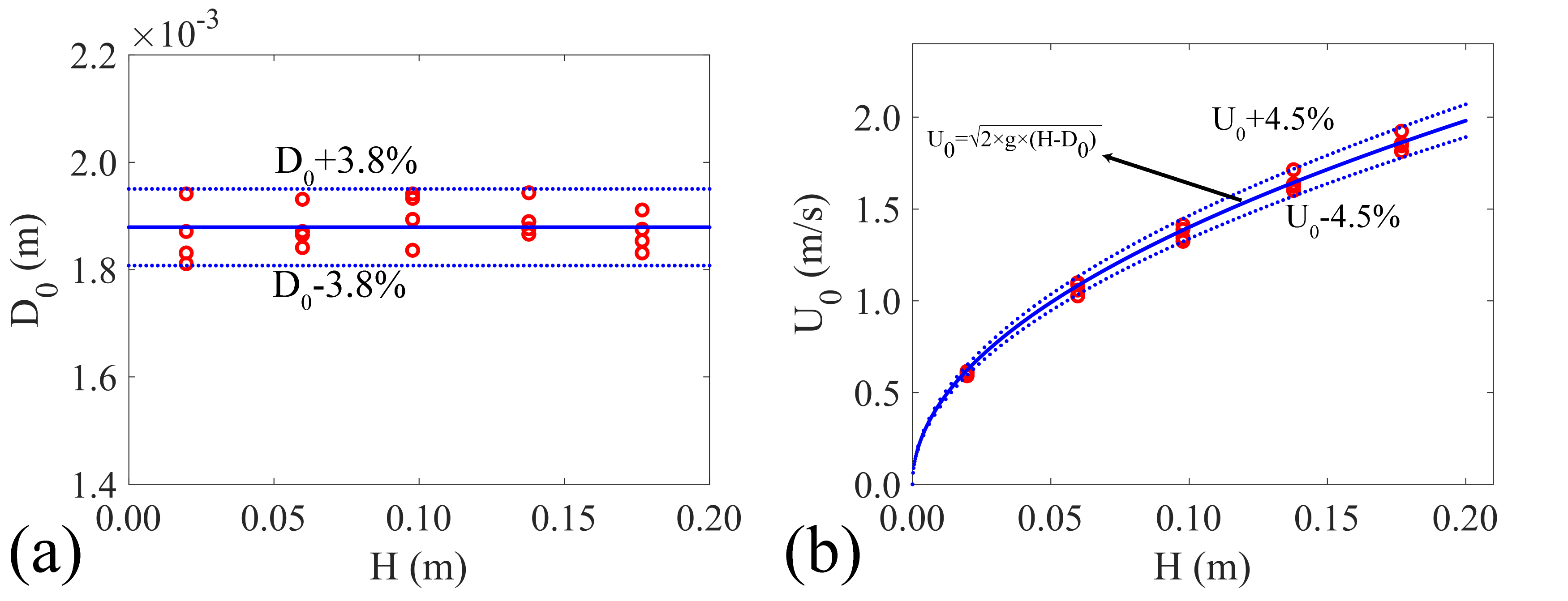

Supplement: Supplementary file 2 — la0c01628_si_002.zip [file la0c01628_si_002.zip › Graphics/Figure S4.tif]

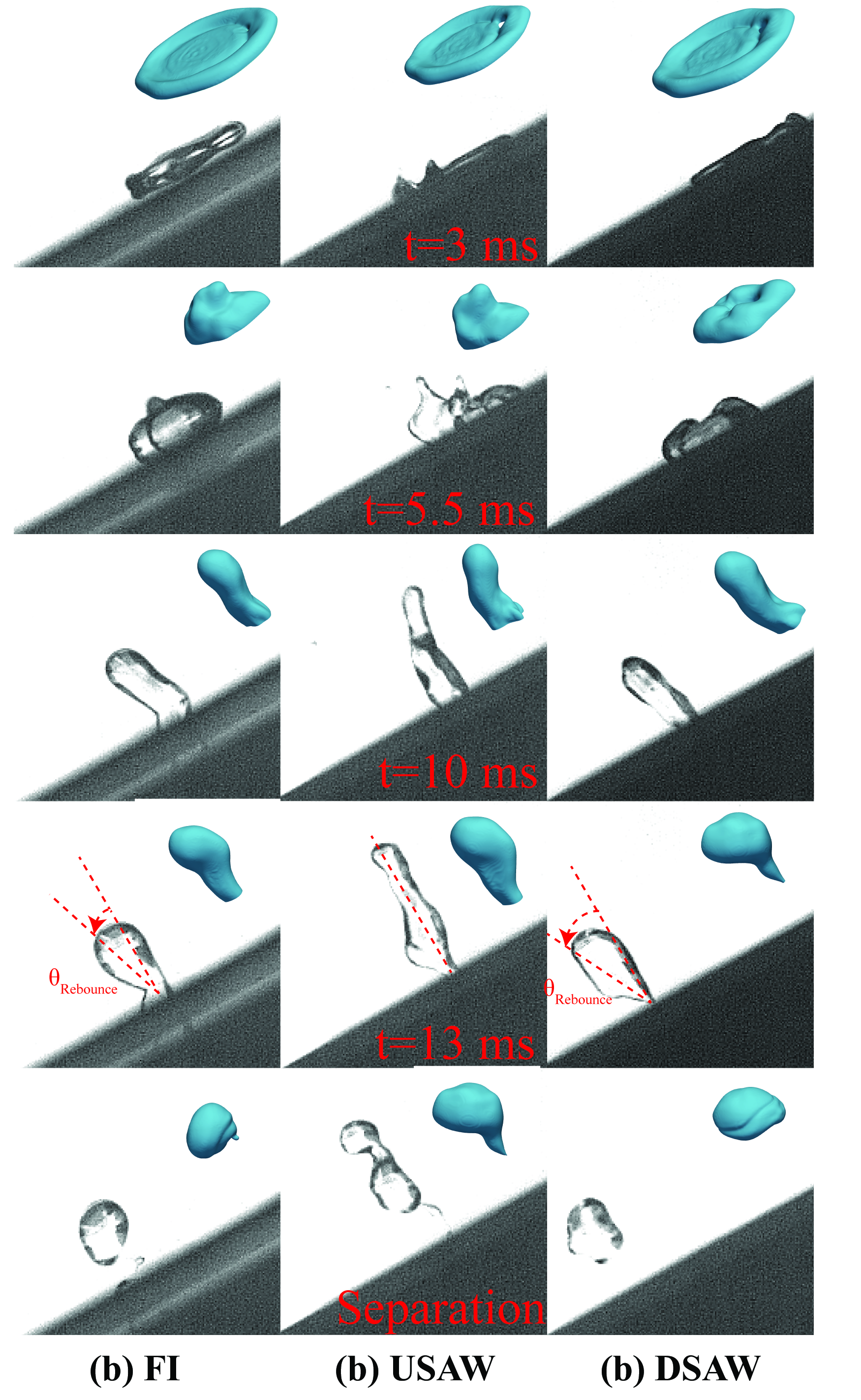

Supplement: Supplementary file 2 — la0c01628_si_002.zip [file la0c01628_si_002.zip › Graphics/Figure S5.tif]

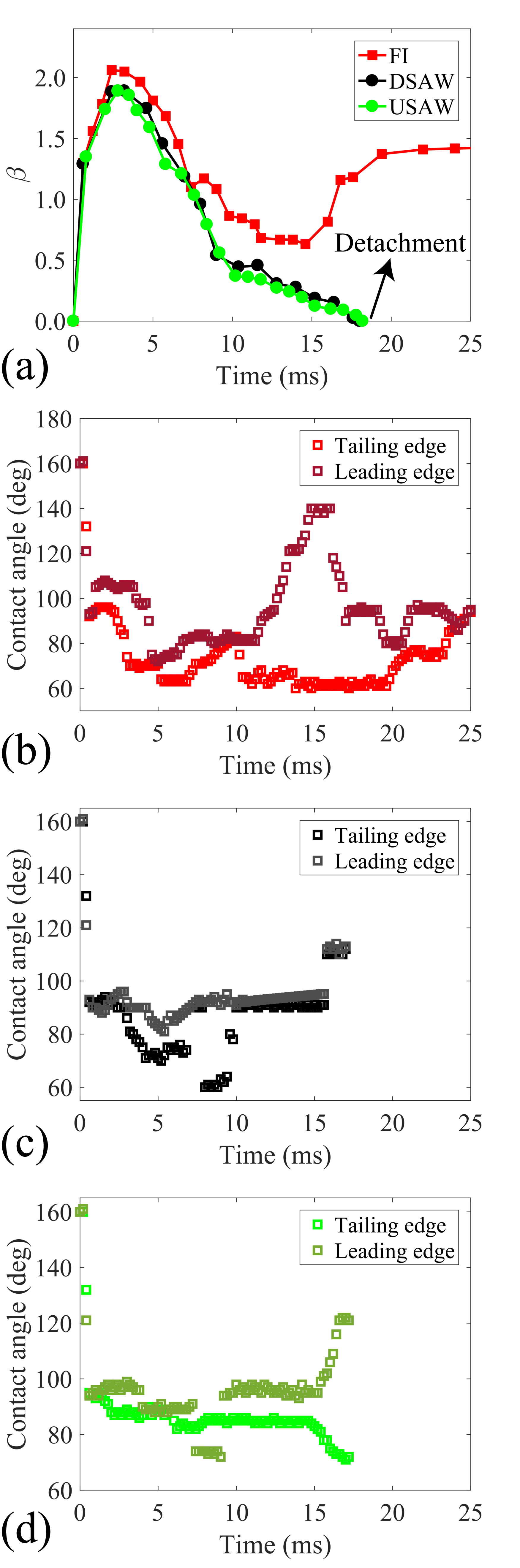

Supplement: Supplementary file 2 — la0c01628_si_002.zip [file la0c01628_si_002.zip › Graphics/Figure S6.tif]

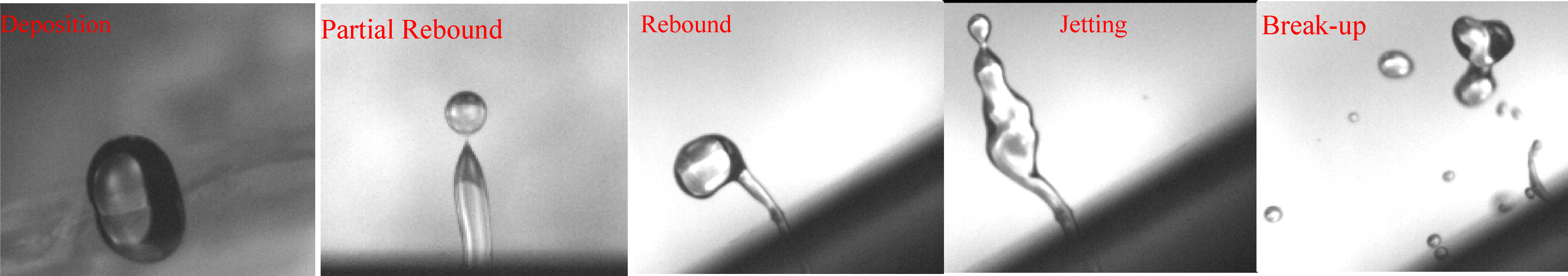

Supplement: Supplementary file 2 — la0c01628_si_002.zip [file la0c01628_si_002.zip › Graphics/Figure S7.tif]

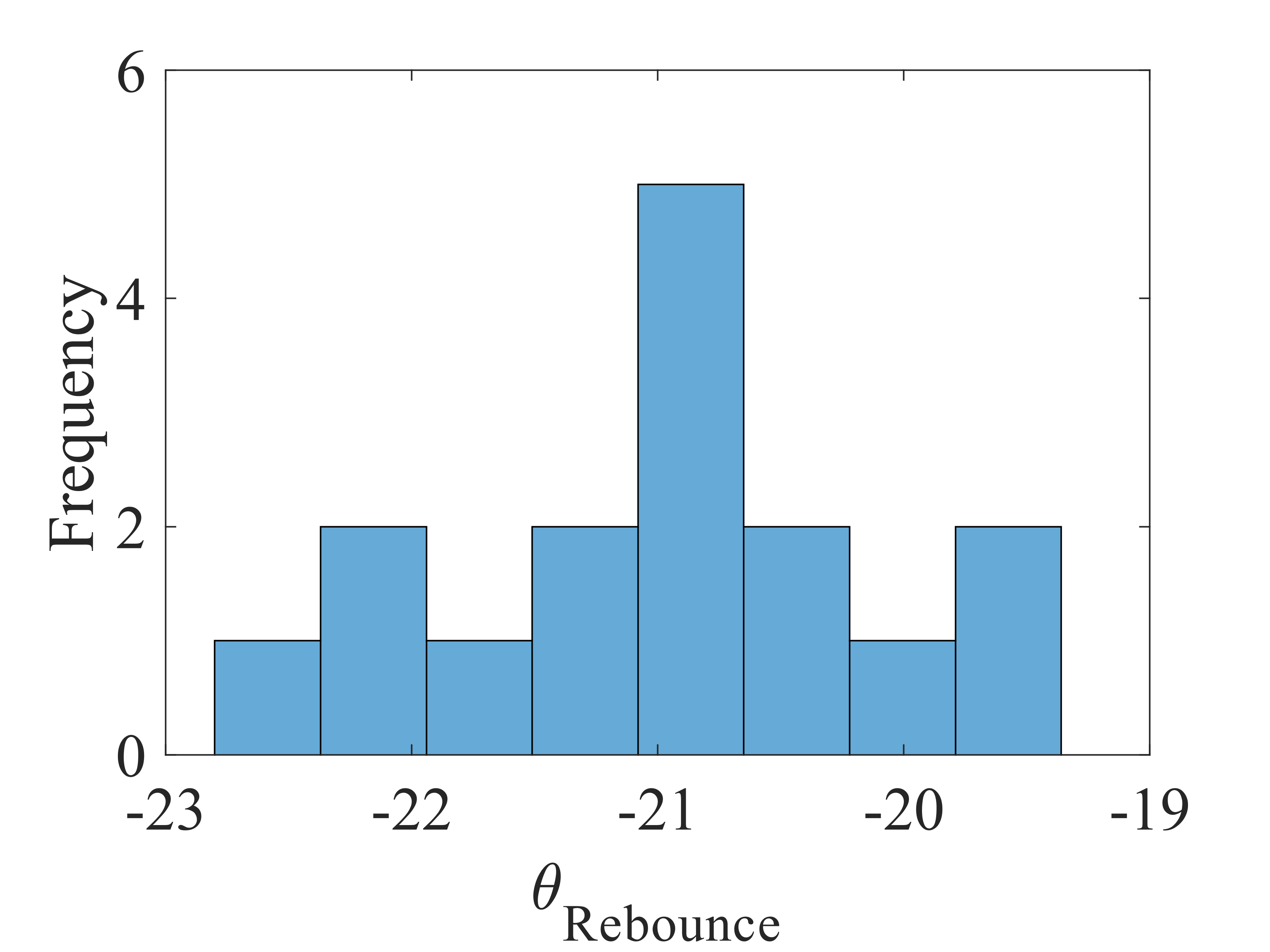

Supplement: Supplementary file 2 — la0c01628_si_002.zip [file la0c01628_si_002.zip › Graphics/Figure S8.tif]

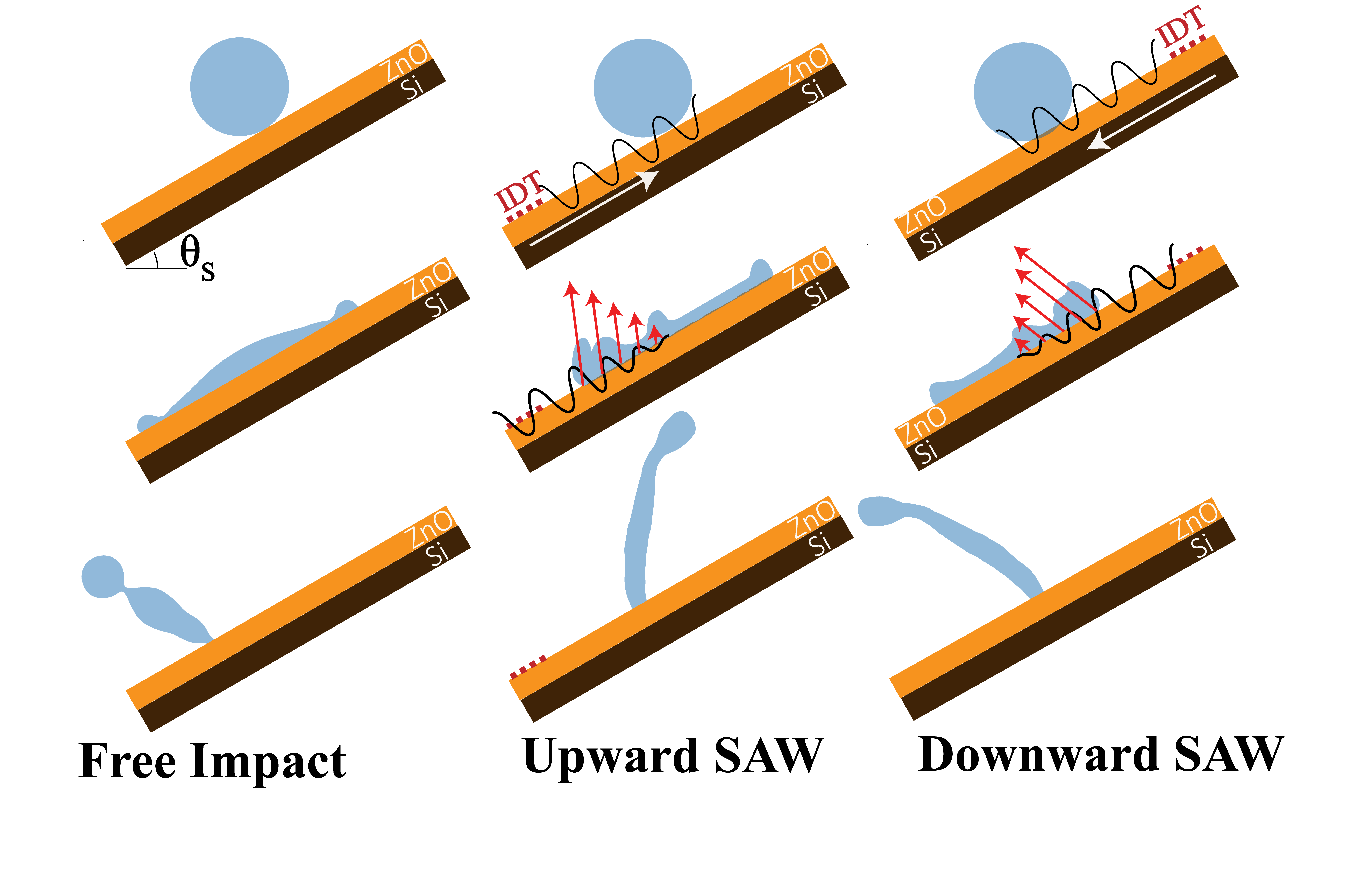

Supplement: Supplementary file 2 — la0c01628_si_002.zip [file la0c01628_si_002.zip › Graphics/Table of Contents Graphic.tif]
